# Supplementary material for: HIV-1 subtype A1, D, and recombinant proviral genome landscapes during long-term suppressive therapy
Source: Nat Commun. 2024 Jul 2;15:5480. doi: 10.1038/s41467-024-48985-9 (PMC11219899; doi:10.1038/s41467-024-48985-9)
Supplement: Supplementary file 1 — Supplementary Information [file 41467_2024_48985_MOESM1_ESM.pdf]

Supplemental Material for:

HIV-1 subtype A1, D, and recombinant proviral genome landscapes during long-term suppressive therapy

Guinevere Q. Lee[1]*, Pragya Khadka[1], Sarah N. Gowanlock[2], Dennis C. Copertino Jr.[1], Maggie C. Duncan[3,4], F. Harrison Omondi[3,4], Natalie N. Kinloch[3,4], Jingo Kasule[5], Taddeo Kityamuweesi[5], Paul Buule[5], Samiri Jamiru[5], Stephen Tomusange[5], Aggrey Anok[5], Zhengming Chen[6], R. Brad Jones[1], Ronald M. Galiwango[5], Steven J. Reynolds[5,7,8], Thomas C. Quinn[7,8], Zabrina L. Brumme[3,4], Andrew D. Redd[7,8,9 †] and Jessica L. Prodger[2,10 †]

[1] Department of Medicine, Division of Infectious Diseases, Weill Cornell Medicine, New York, NY, USA

[2] Department of Microbiology and Immunology, Western University, London, ON, Canada.

[3] Faculty of Health Sciences, Simon Fraser University, Burnaby, BC, Canada

[4] British Columbia Centre for Excellence in HIV/AIDS, Vancouver, BC, Canada

[5] Rakai Health Sciences Program, Kalisizo, Uganda

[6] Department of Population Health Sciences, Division of Biostatistics, Weill Cornell Medicine, New York, NY, USA

[7] Division of Intramural Research, National Institute of Allergy and Infectious Diseases, National Institutes of Health, Bethesda, MD, USA

[8] Department of Medicine, Johns Hopkins University School of Medicine, Baltimore, MD, USA

[9] Institute of Infectious Disease and Molecular Medicine, University of Cape Town, South Africa

[10] Department of Epidemiology and Biostatistics, Western University, London, ON, Canada.

* Corresponding author: Guinevere Q. Lee, PhD; gul4001@med.cornell.edu

[†] ADR and JLP contributed equally as co-senior authors

Table of contents

Figure S1. Full-genome HIV subtyping of donor proviruses by FLIP-seq and MOCHI
Donor 1....4
Donor 2....5
Donor 3....6
Donor 4....7
Donor 5....8
Donor 6....9
Donor 7....10
Donor 8....11
Donor 9....12
Donor 10....13
Donor 11....14
Donor 13....15
Donor 14....16
Donor 14....17
Donor 15....18
Donor 16....19
Donor 17....20
Donor 18....21
Donor 19....22
Donor 20....23
Donor 21....24
Donor 22....25
Donor 23....26

Figure S2. Commonly deleted HIV regions in RHSP cohort proviruses....27

Figure S3. Inferred viral intactness as determined by IPDA *psi* and *env* primer and probe binding locations for subtypes A1, D and recombinants....28

Figure S4. Sequence diversity within the IPDA *psi* and *env* primer/probe regions in the RHSP cohort....29

Figure S5. Comparison of IPDA-B and IPDA-A1D....30

Figure S6. IPDA-A1D applied to a donor without HIV (negative control)....31

Figure S7. IPDA-A1D hypermutation discrimination validation....32

Figure S8. Examination of the resilience of *IPDA-B* probes to the type of within-host HIV diversity seen in Donor 20....33

Table S1. Donor characteristics....35

Table S2. Reservoir measurements....36

Table S3. Detailed breakdown of HIV DNA genome defectiveness categories, count data....37

Table S4. Genome categories by subtype, count data..38

Table S5. Detailed breakdown of clonal sequences, count data..................................39

Table S6. Detailed breakdown of clonal sequences, by subtype................................40

Table S7. Count of hypermutated versus non-hypermutated genomes among all the defective genomes that are IPDA *psi*+*env*+..41

Table S8. Original (IPDA-B) and modified (IPDA-A1D) primer and probe sequences...42

Table S9. Four donors failing IPDA-B were rescued by IPDA-A1D...............................43

Supplemental section: ddPCR and FLIP-seq primer/probe validation.........................44

Table S10. ddPCR (total HIV DNA) and FLIP-seq primers and probes.............45

Figure S9. Comparison of ddPCR primers and probe to HIV subtype reference sequences (QuickAlign output)...46

Figure S10. Comparison of FLIP-seq primers to HIV subtype reference sequences (QuickAlign output)...47

Supplemental section: Validation of HIVSeqinR for HIV subtypes A1 and D..............49

Figure S11. Published HIVSeqinR decision tree for classification of viral genomes as defective versus intact..50

Figure S12. Lengths of discordantly classified contigs when different BLASTN reference sequences were used..51

Figure S13. HIV protein lengths in the Rakai cohort..52

Figure S14. *psi* region alignments of 18 QVOA viral RNA genomes categorized as intact and 18 DNA genomes categorized as 5' defective...............................54

Figure S15. HIVSeqinR is predicts subtype A1 and D genome intactness with 83% sensitivity...54

Figure S16. HIV-1 subtyping of the 18 QVOA-derived RNA genomes by MOCHI Proviral Subtyping Express 1.0 reveals that 5' defect was associated with both subtype A1 and D genomes at the 5' end...55

References for this section...57

.

Donor 1 – subtype D

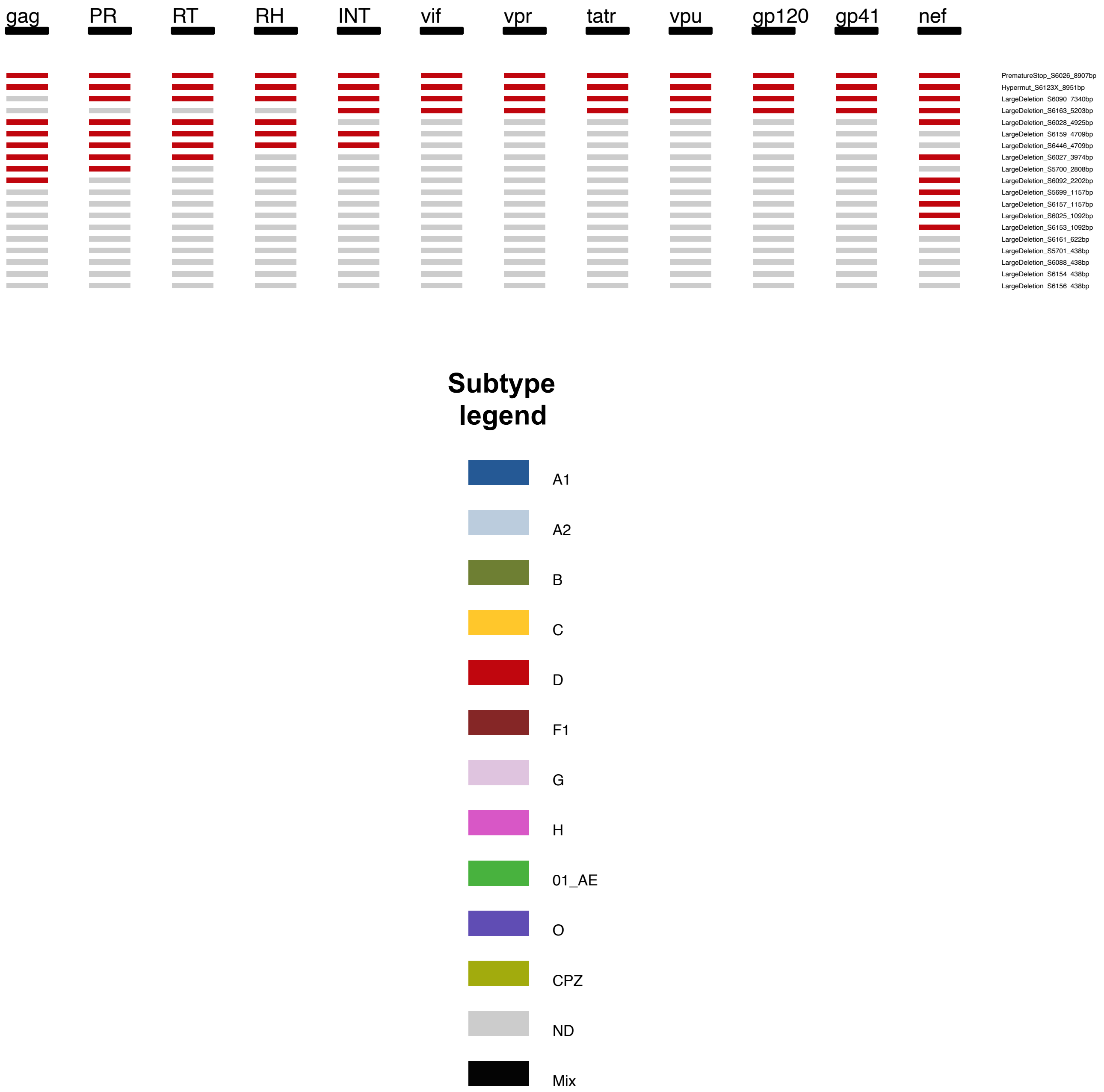

Figure S1: Full-genome HIV subtyping by FLIP-seq and MOCHI: Donor 1. The best-matching HIV subtype for each viral gene region is denoted by its color. HIV genomic regions are not drawn to scale. Donor 1's viral subtype is D.

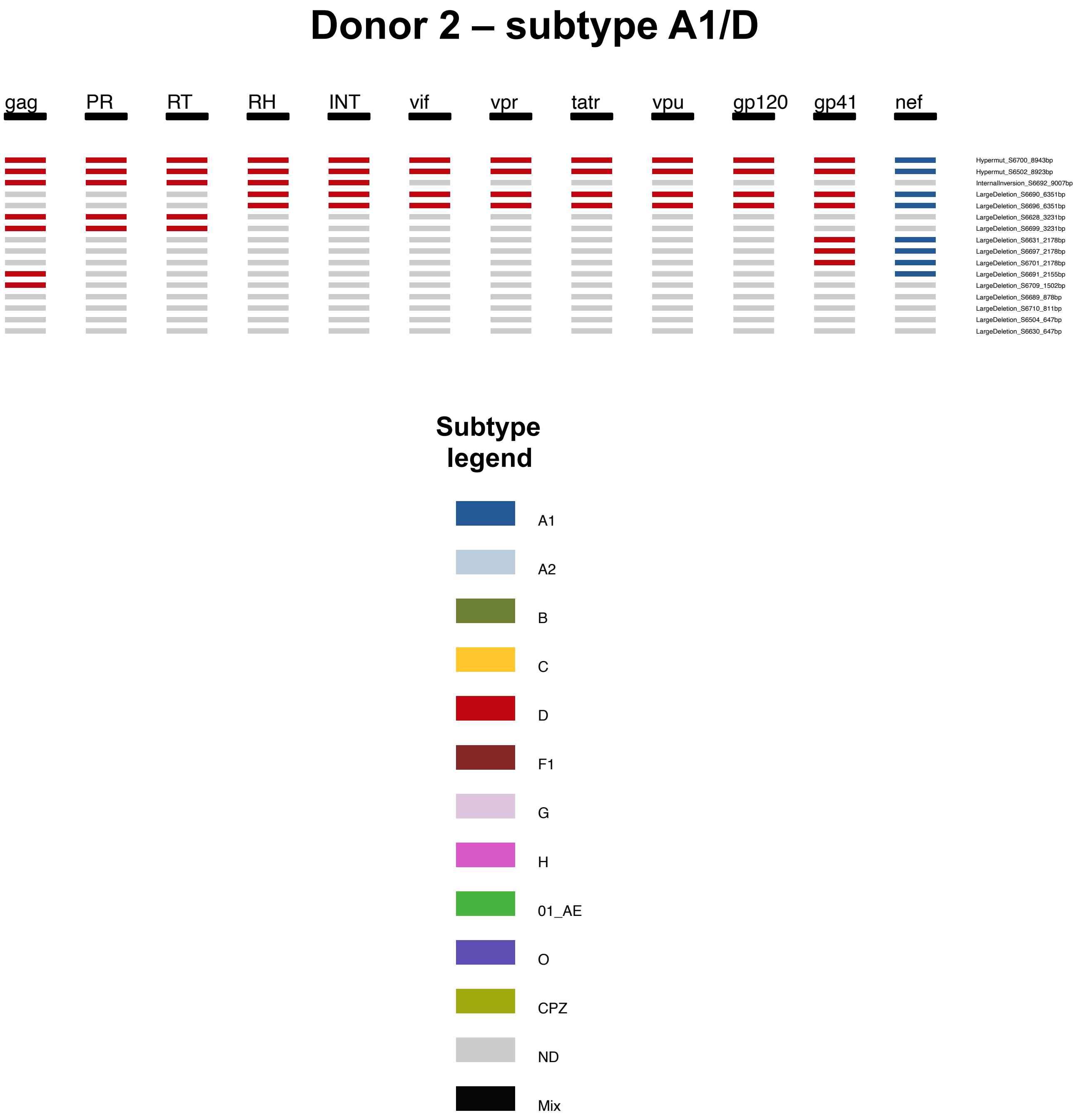

Figure S1: Full-genome HIV subtyping by FLIP-seq and MOCHI: Donor 2. Donor 2’s viral subtype was designated as A1/D recombinant, where the transition between A1 and D genomic regions occurs somewhere near the end of *gp41* and the beginning of *nef*.

Donor 3 – subtype A1

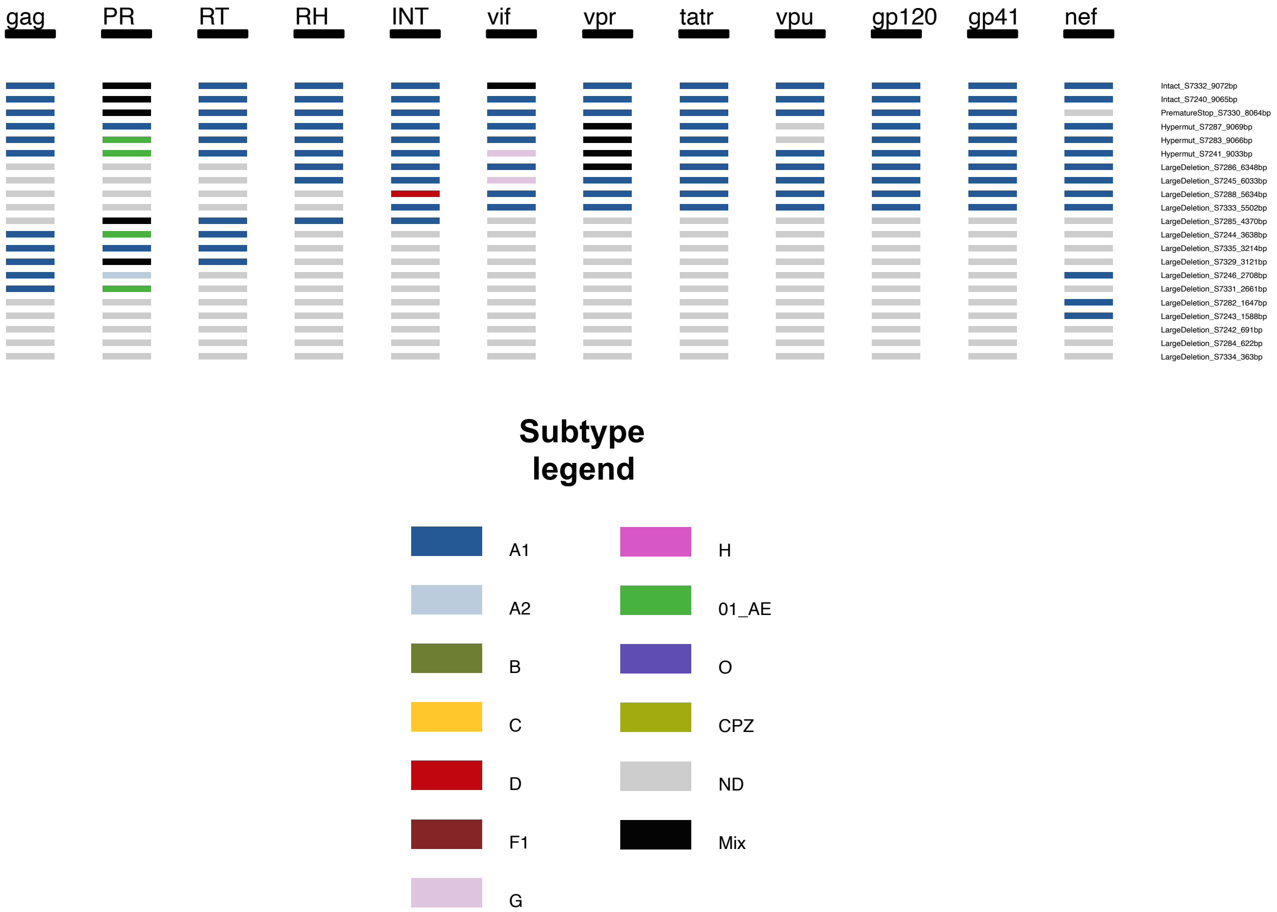

Figure S1: Full-genome HIV subtyping by FLIP-seq and MOCHI: Donor 3.
When interpreting HIV subtype data, it is important to keep in mind that the assigned subtype for a given HIV region simply represents the subtype of the HIV reference sequence that best matched the query sequence in that HIV region. Some HIV regions however, particularly those that are short and/or highly conserved, do not contain sufficient genetic information to make subtype calls with high confidence. Donor 3 is an example of such a case, which requires some manual interpretation.

Though four of donor 3's protease sequences best matched CRF01_AE, the 5' region of CRF01_AE is subtype A1, and therefore it cannot always be discriminated with high confidence from A1, particularly if the query region is short. Given that the donor's other protease sequences are A1, and CRF01_AE is rare in Uganda, these three calls were interpreted as likely subtype A1. Similarly, though a single integrase sequence returned subtype D as the best-match, this integrase sequence was only partial, and scored the best match by only 0.1% (78.3% for D versus 78.2% for A1). Given that all of the donor's other integrase sequences are A1, we interpreted this sequence as likely A1 as well. Finally, though two of the donor's *vif* sequences best matched subtype G, subtype A1 was a close second by only 0.2%. Given this, and *vif*'s short length, we also interpreted these as likely A1. Donor 3's viral subtype was thus designated as A1.

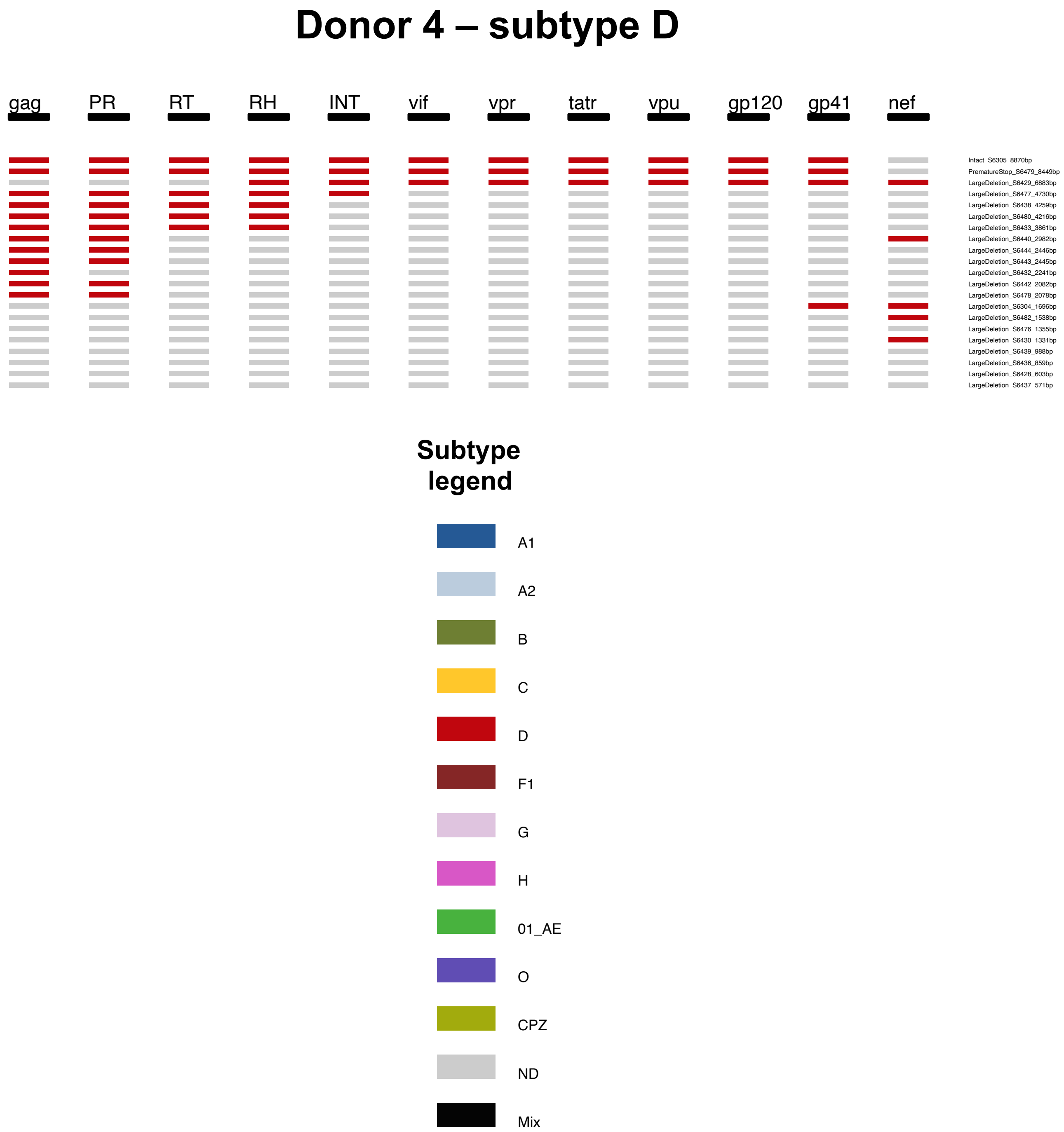

Figure S1: Full-genome HIV subtyping by FLIP-seq and MOCHI: Donor 4. Donor 4’s viral subtype is D.

Donor 5 – subtype D

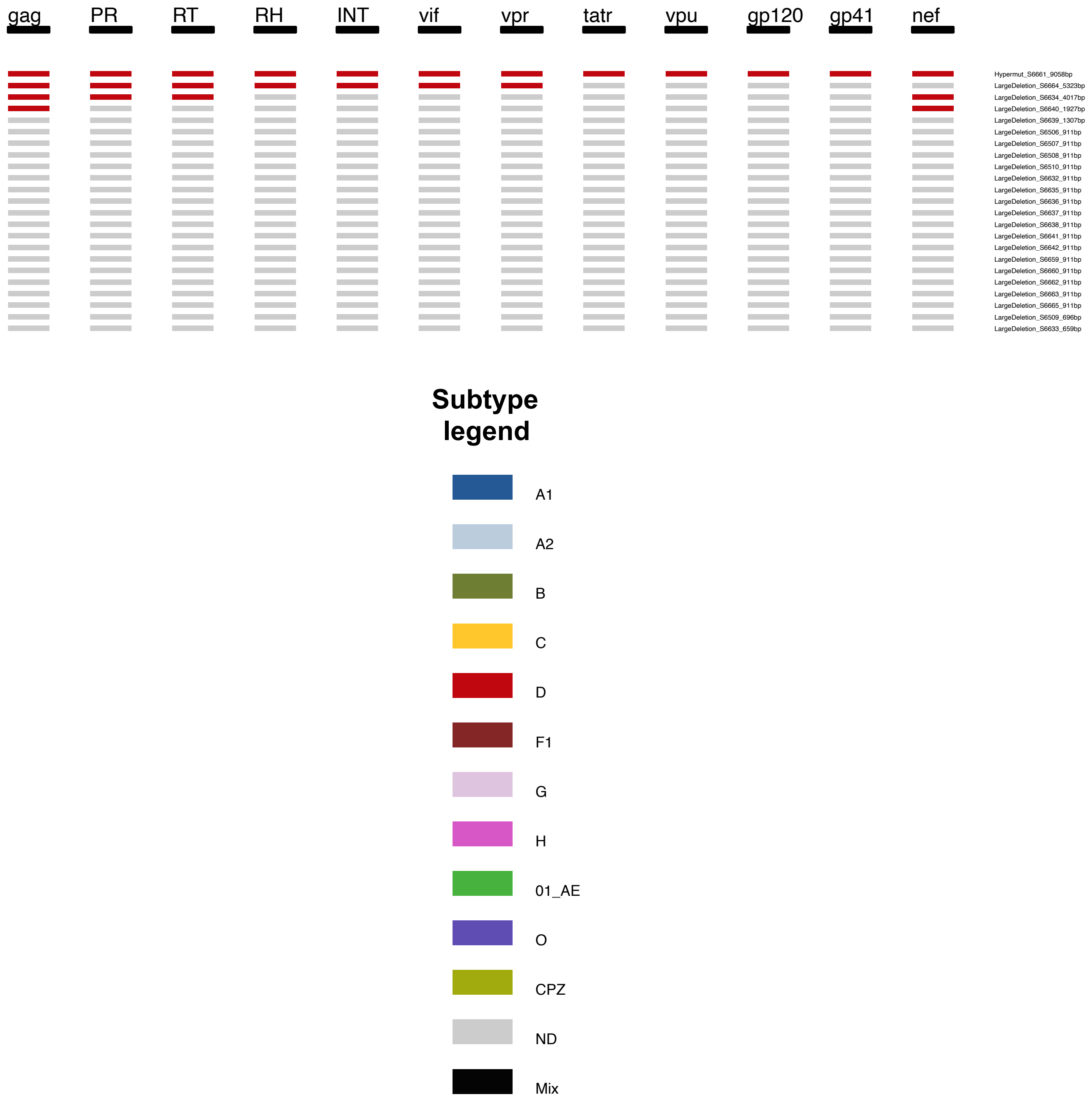

Figure S1: Full-genome HIV subtyping by FLIP-seq and MOCHI: Donor 5.
Donor 5’s viral subtype is D.

Donor 6 – subtype A1/D

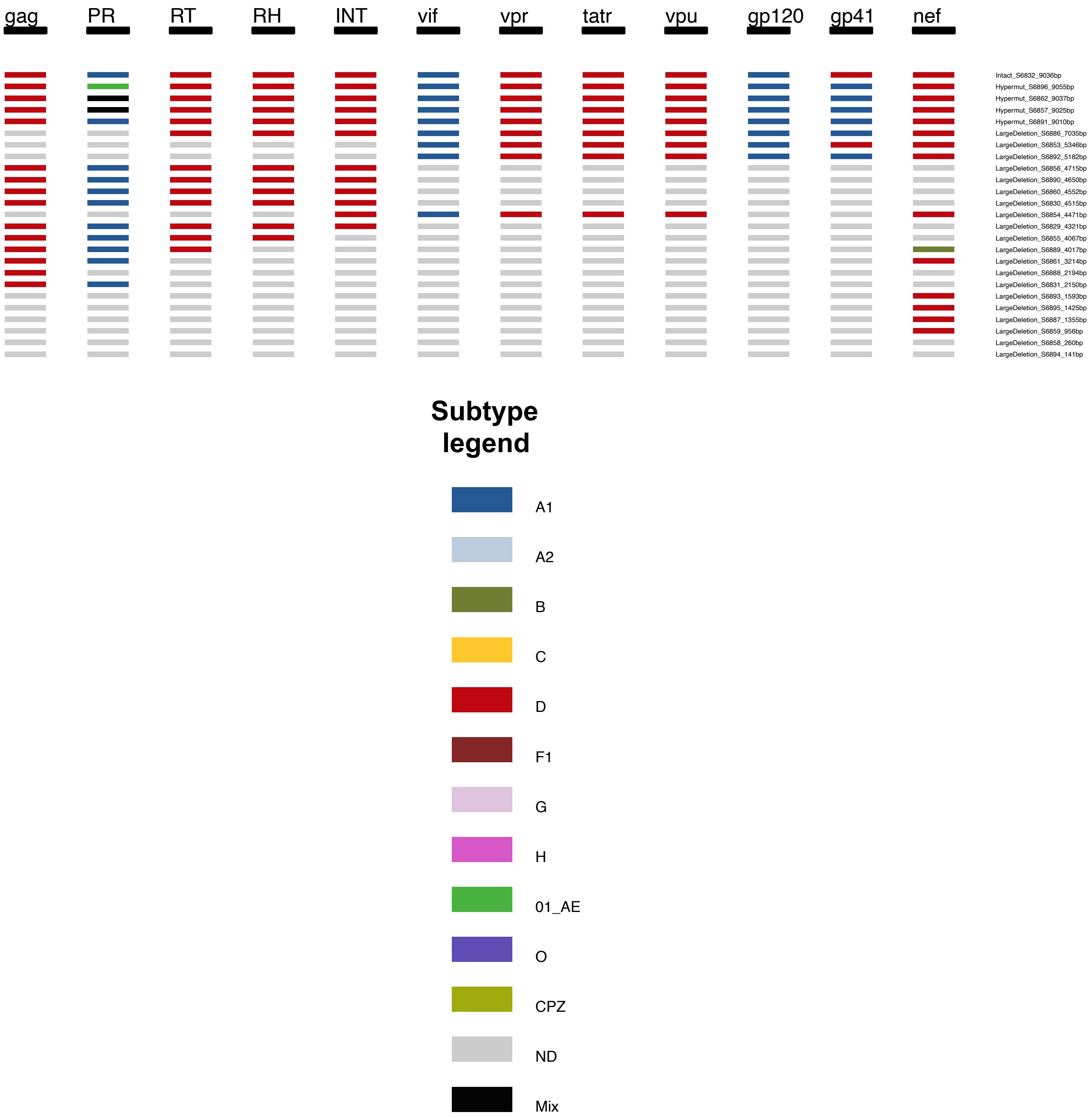

Figure S1: Full-genome HIV subtyping by FLIP-seq and MOCHI: Donor 6. The single CRF01_AE subtype call in protease was interpreted as likely A1, as CRF01_AE is subtype A1 in this genomic region. Similarly, the single subtype B call in *nef* was interpreted as likely subtype D. This is because subtypes B and D are closely related, and D is far more likely, given that subtype B is rare in Uganda and the donor's other *nef* sequences were classified as subtype D. Donor 6's viral subtype was designated as an A1/D recombinant, which features a complex mosaic of A1 and D regions.

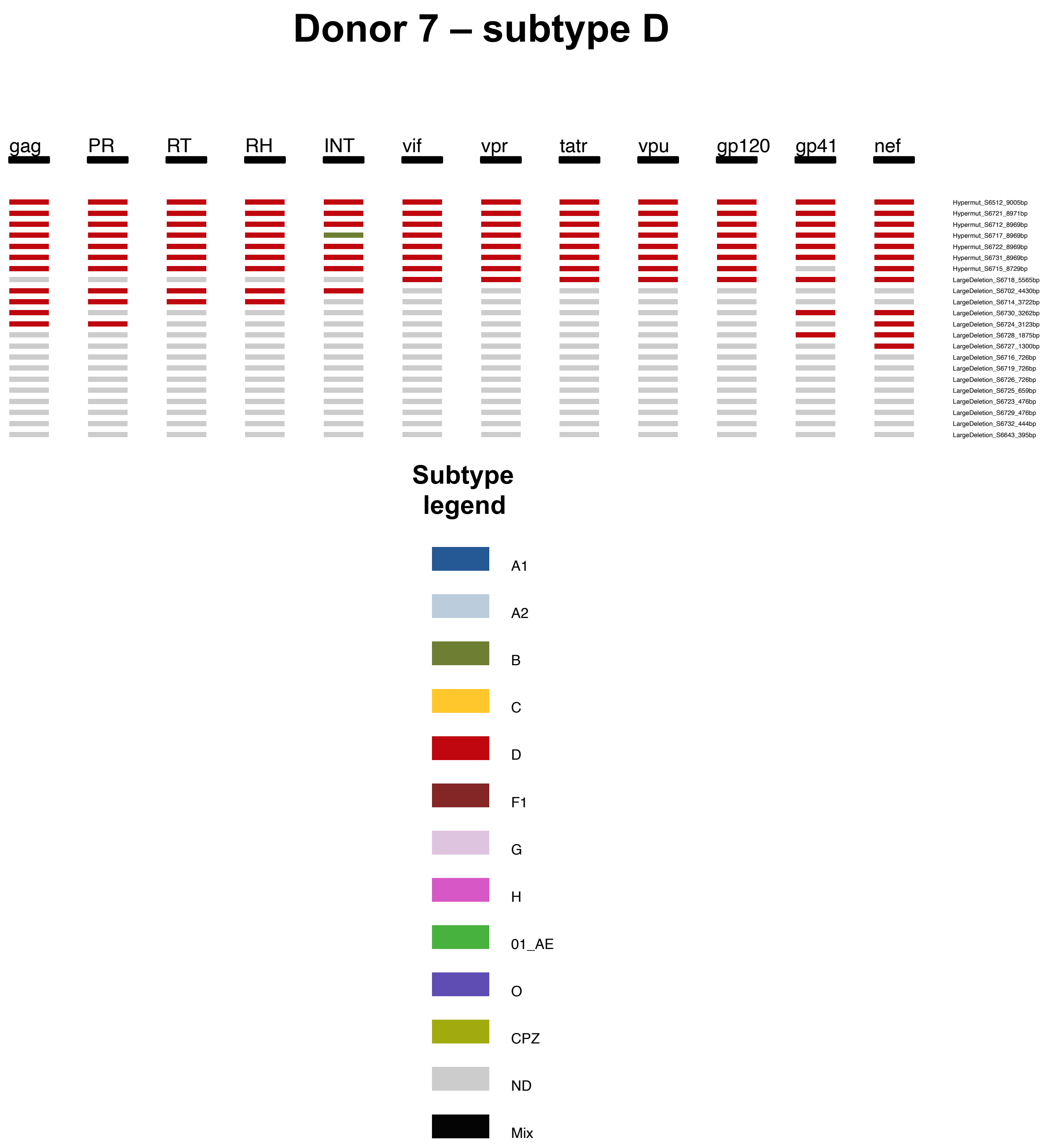

Figure S1: Full-genome HIV subtyping by FLIP-seq and MOCHI: Donor 7. Donor 7's viral subtype is D.

Donor 8 – subtype A1/D

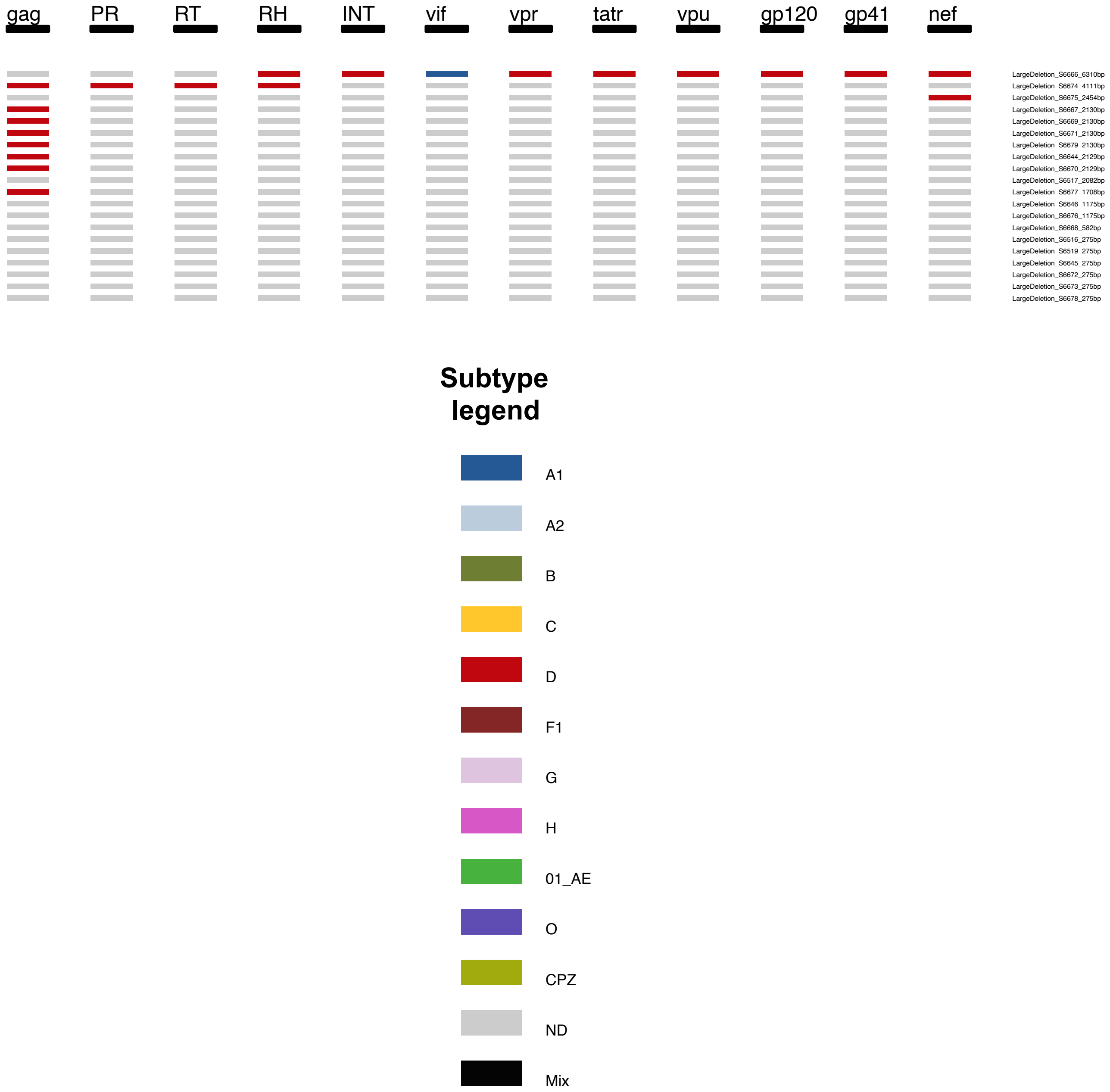

Figure S1: Full-genome HIV subtyping by FLIP-seq and MOCHI: Donor 8.
Though only one of Donor 8's sequences had coverage within *vif*, it matched the subtype A1 reference with 93.9% similarity in this region (compared to only 89.4% similarity to the subtype D reference). Donor 8's viral subtype was therefore designated as A1/D recombinant, where the region surrounding *vif* is subtype A1 whereas the rest of the genome is D.

Donor 9 – subtype D

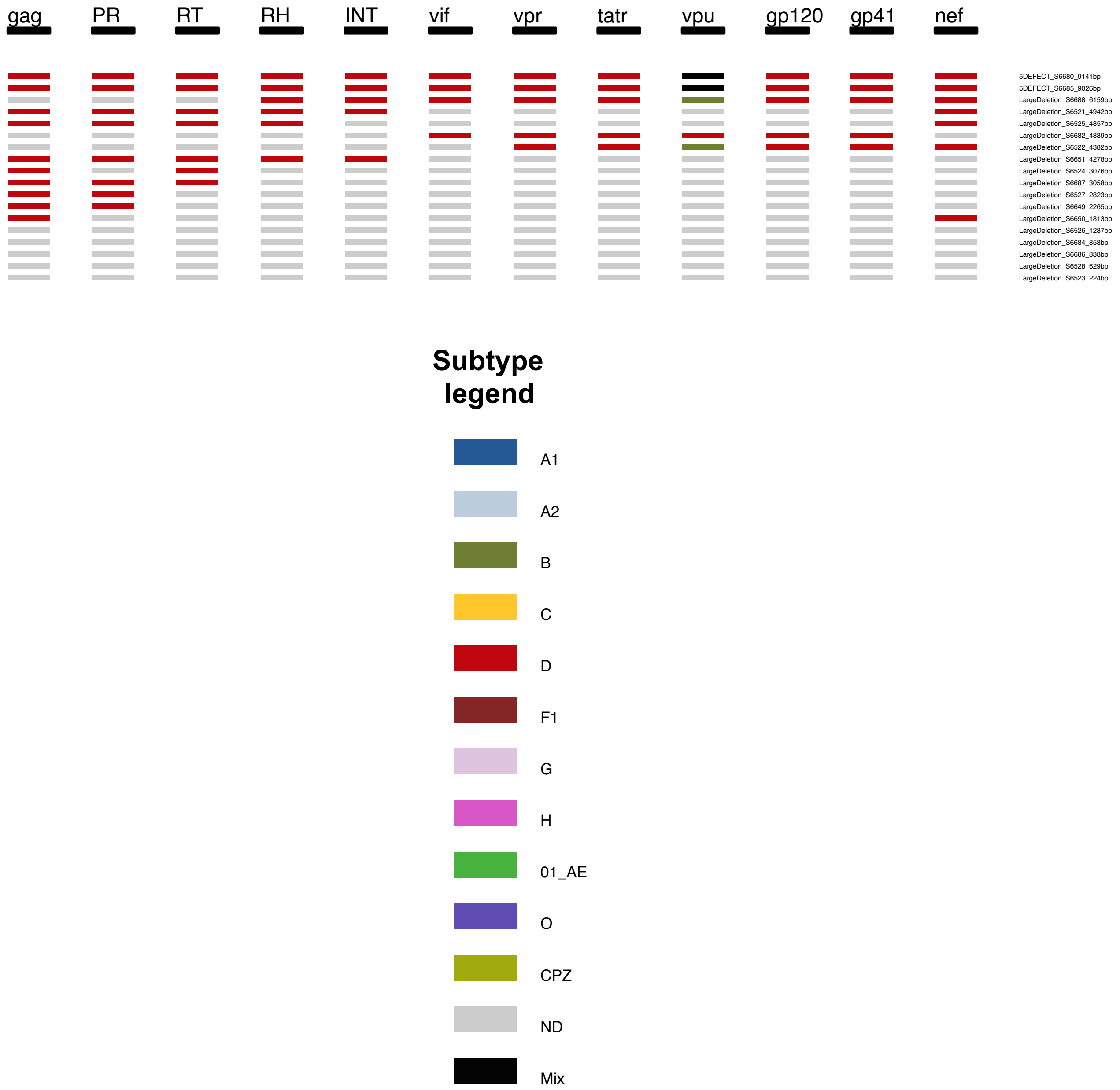

Figure S1: Full-genome HIV subtyping by FLIP-seq and MOCHI: Donor 9. Though two of donor 9's *vpu* sequences returned subtype B, these were interpreted as subtype D because of 1) the close relatedness between subtypes B and D , 2) the rarity of B in Uganda and 3) the fact that another *vpu* sequence rom this donor returned a subtype D call. Donor 9 was thus classified as subtype D.

Donor 10 – subtype D

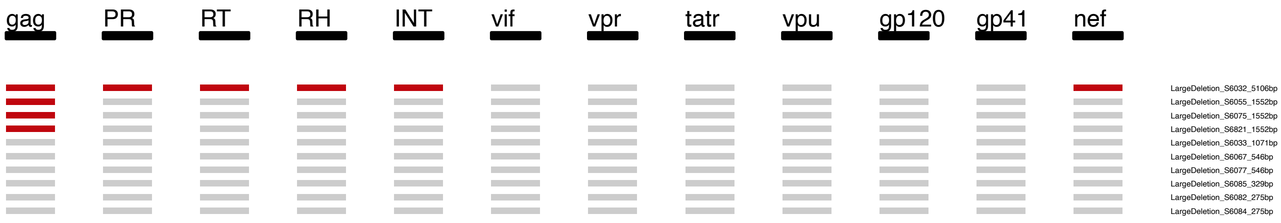

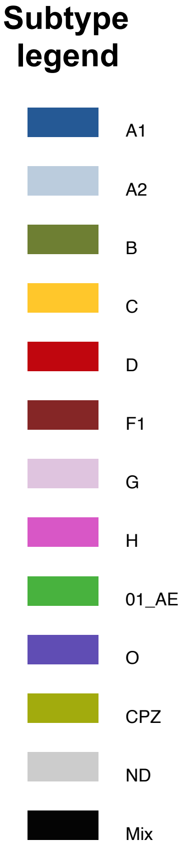

Figure S1: Full-genome HIV subtyping by FLIP-seq and MOCHI: Donor 10. Donor 10's viral subtype is D.

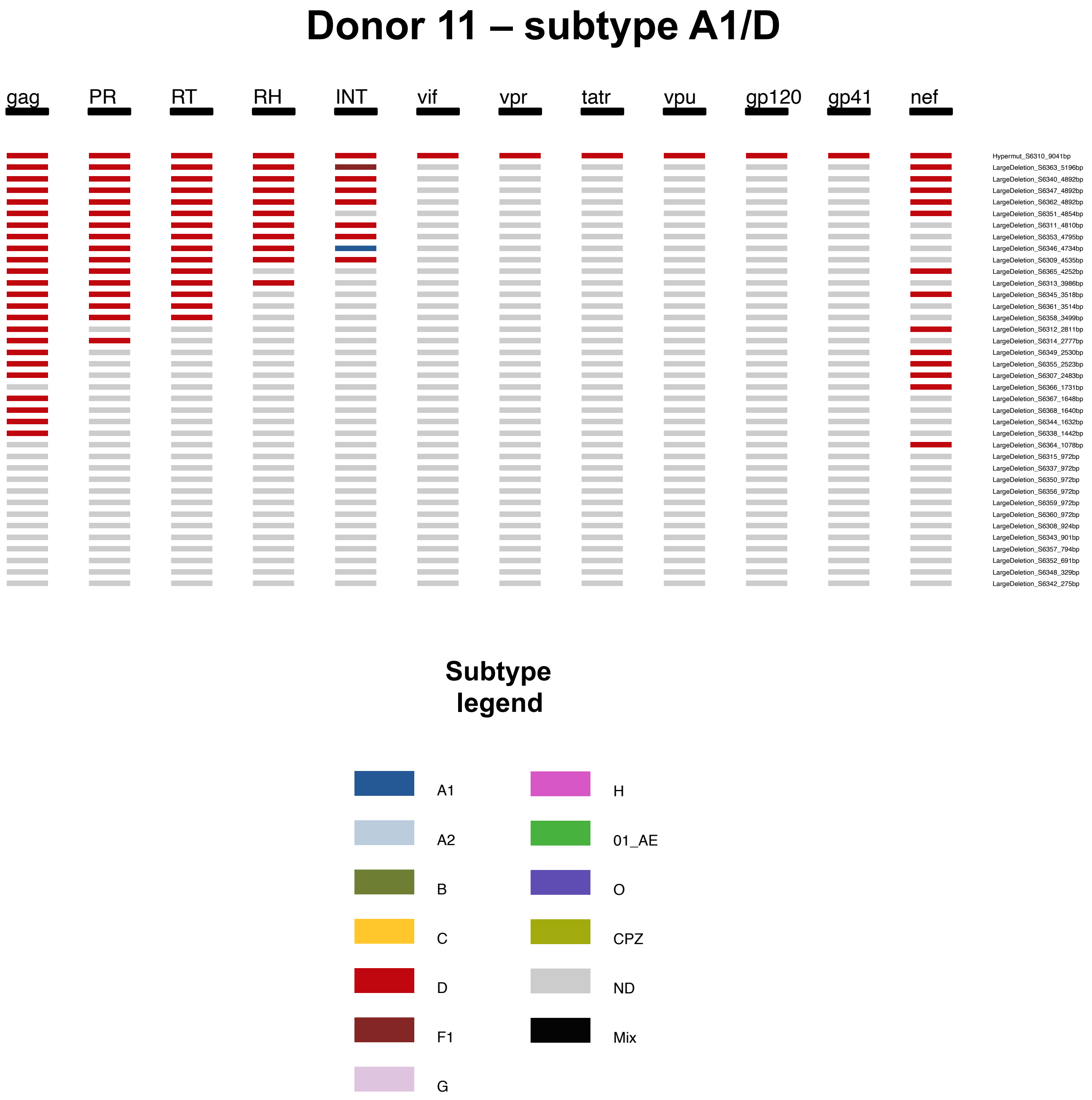

Figure S1: Full-genome HIV subtyping by FLIP-seq and MOCHI: Donor 11.
Donor 11's viral subtyping result was somewhat ambiguous. All regions returned subtype D calls, except for one integrase sequence that returned a subtype F1 call, and another that returned a subtype A1 call. The subtype F1 integrase sequence scored the best match by only 0.1%, where the next-best call was subtype D. Given the rarity of subtype F1 in Uganda, and the fact that most of this donor's other integrase sequences were subtype D, the subtype F1 call was interpreted as likely D. In contrast, the subtype A1 integrase call was less straightforward to interpret as another subtype, as 853 of the 864 bases in this sequence mapped to subtype A1. Given the high prevalence of subtype A1, D and A1/D recombinants in Uganda, and our wish to err on the side of caution when classifying a sequence as a "pure" subtype, this donor was putatively classified as having an A1/D recombinant infection for the purposes of this study. We acknowledge the possibility however that their infection could be subtype D only.

Donor 12 – subtype A1

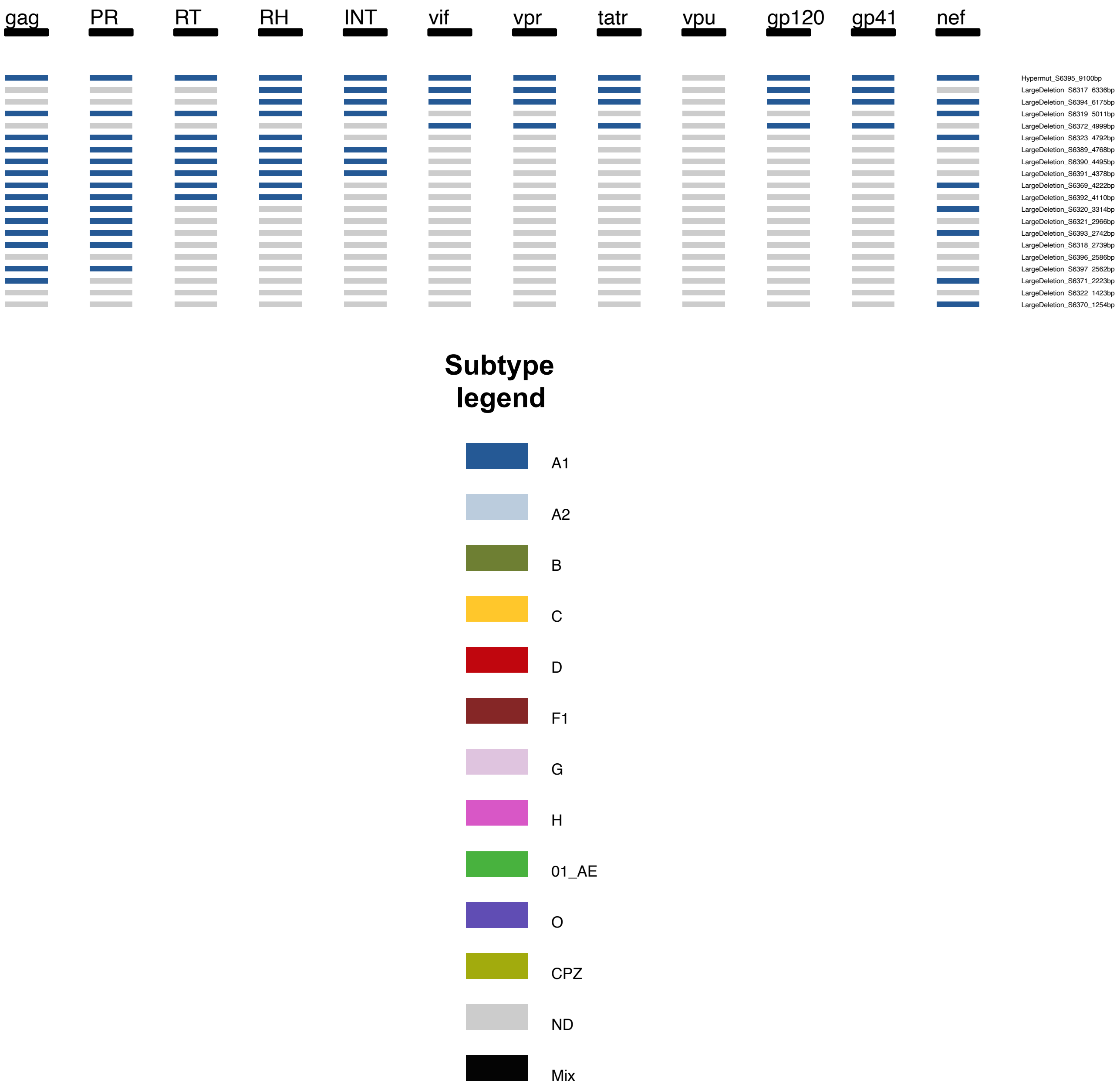

Figure S1: Full-genome HIV subtyping by FLIP-seq and MOCHI: Donor 12. Donor 12's viral subtype is A1.

Donor 13 – subtype A1

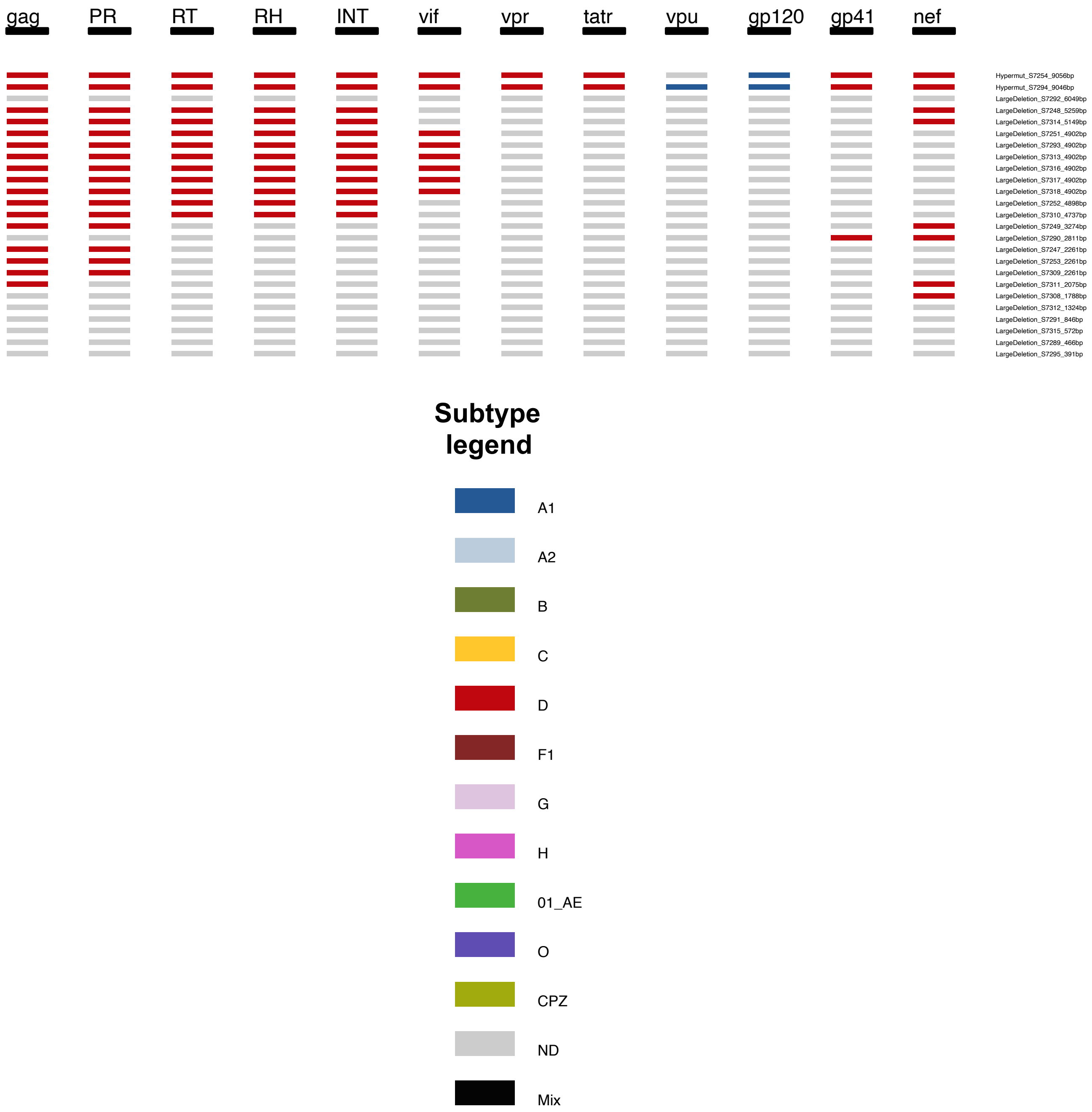

Figure S1: Full-genome HIV subtyping by FLIP-seq and MOCHI: Donor 13. Donor 13's viral subtype was designated as A1/D recombinant, where the *vpu* and *gp120* regions are subtype A1 and the remainder of the genome is subtype D.

Donor 14 – subtype D

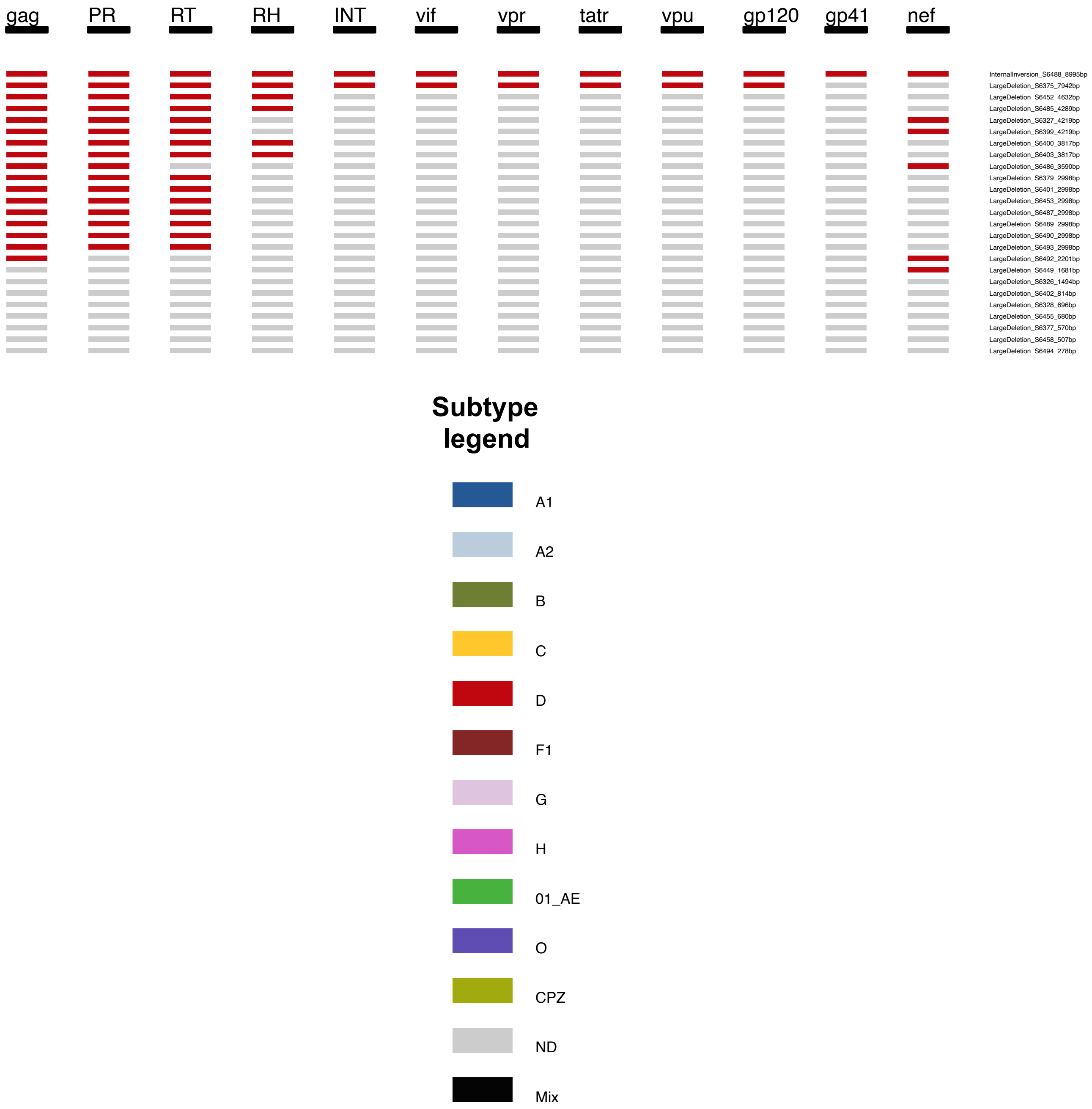

Figure S1: Full-genome HIV subtyping by FLIP-seq and MOCHI: Donor 14. Donor 14's viral subtype is D.

Donor 15 – subtype D

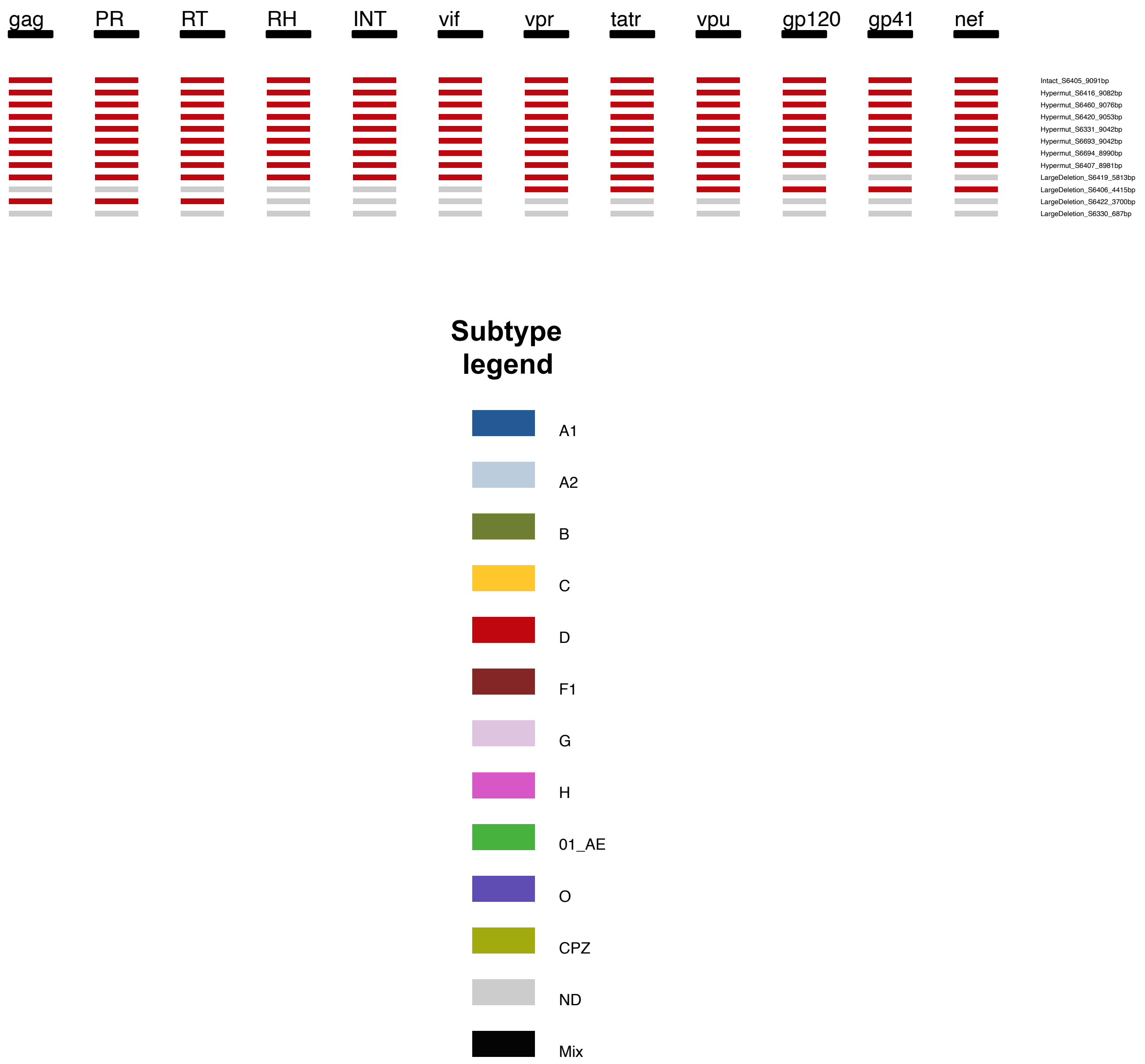

Figure S1: Full-genome HIV subtyping by FLIP-seq and MOCHI: Donor 15. Donor 15's viral subtype is D.

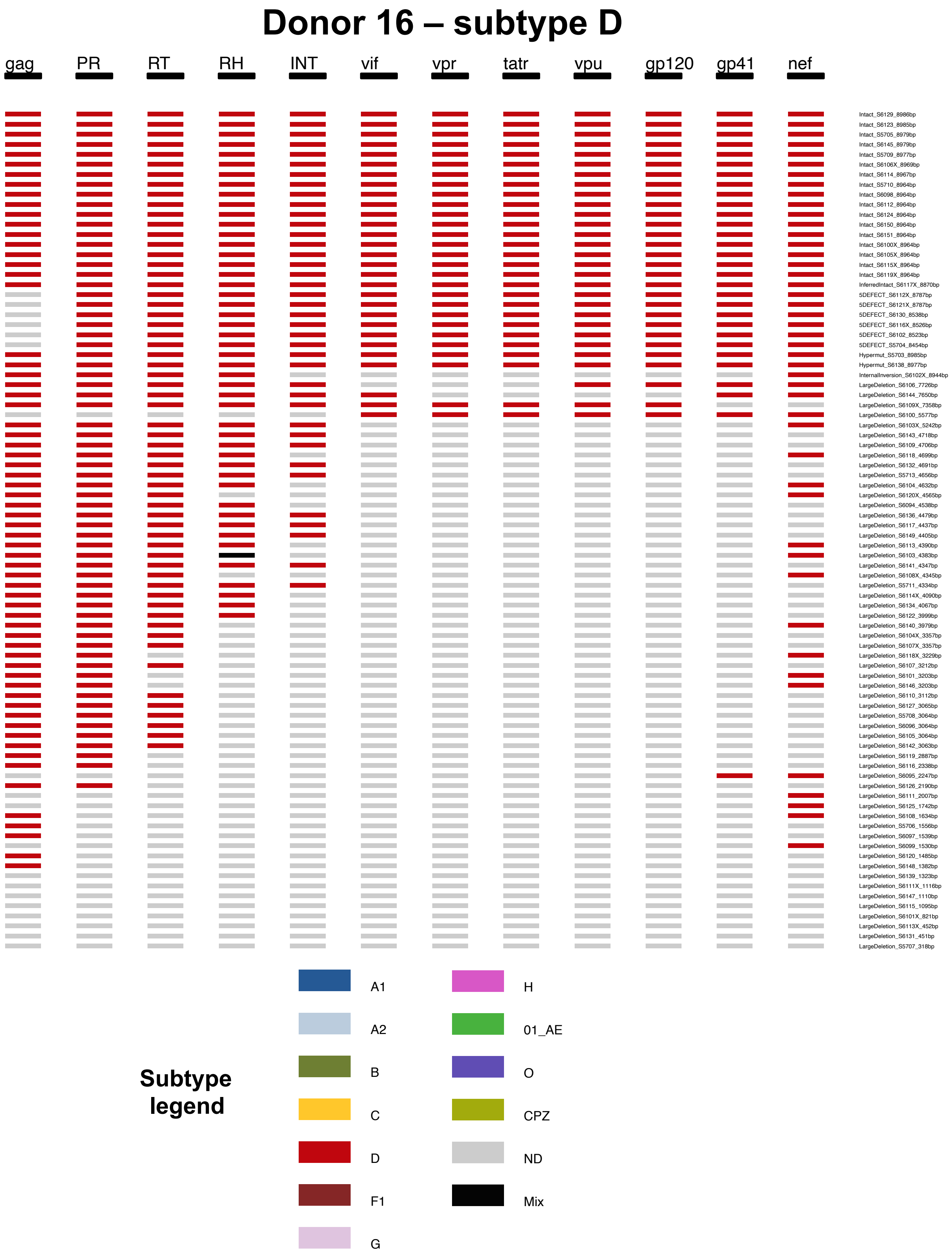

Figure S1: Full-genome HIV subtyping by FLIP-seq and MOCHI: Donor 16.
Donor 16's viral subtype is D.

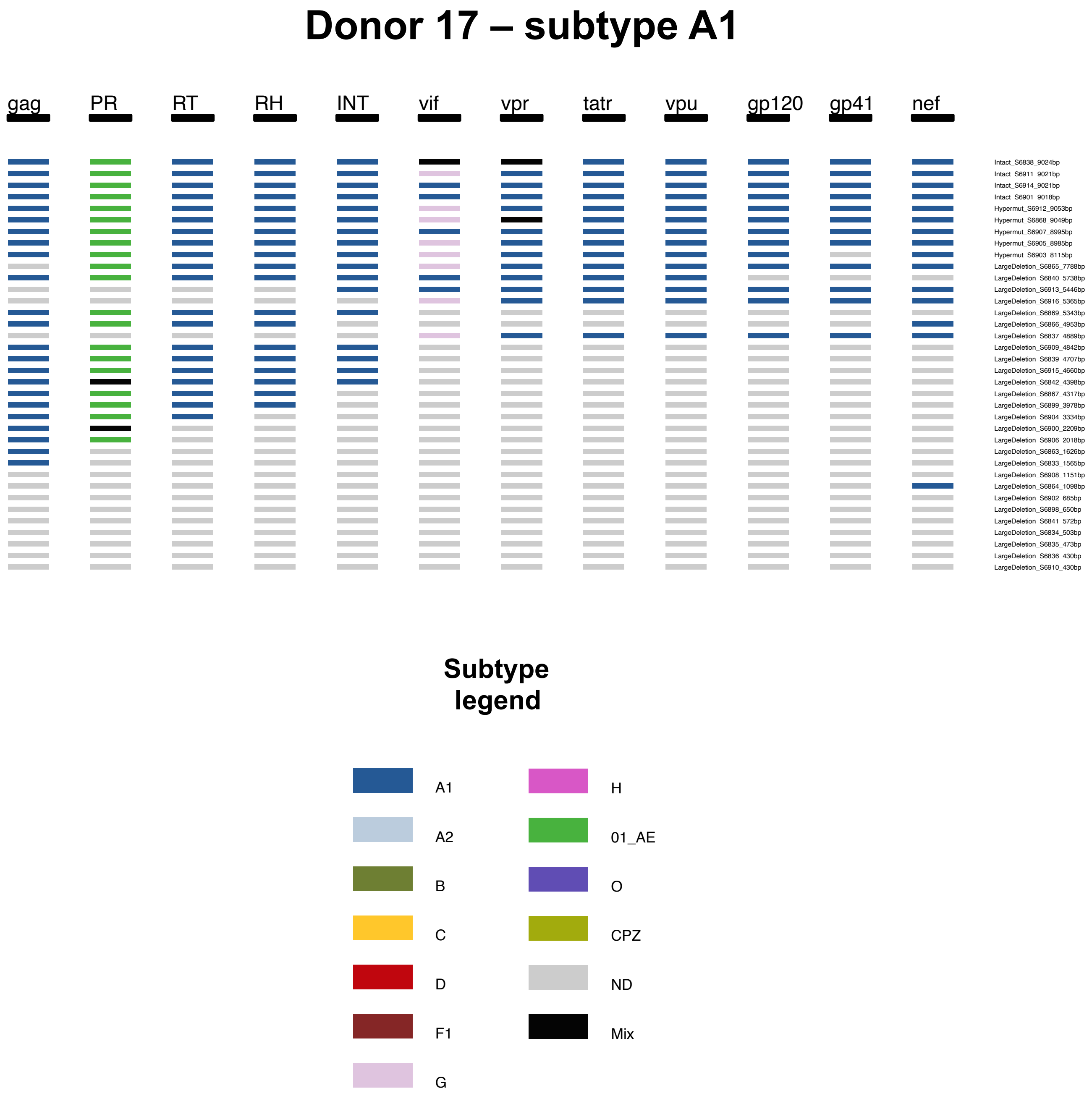

Figure S1: Full-genome HIV subtyping by FLIP-seq and MOCHI: Donor 17.
The CRF01_AE subtype calls in protease was interpreted as likely A1, as CRF01_AE is subtype A1 in this genomic region. Similarly, given the strong subtype A1 signal throughout the genome, the subtype G calls in *vif* were also interpreted as likely subtype A1, as *vif*'s short length does not allow high-confidence subtyping, and subtype G is rare in Uganda. Donor 17's viral subtype was thus designated as A1.

Donor 18 – subtype A1/D

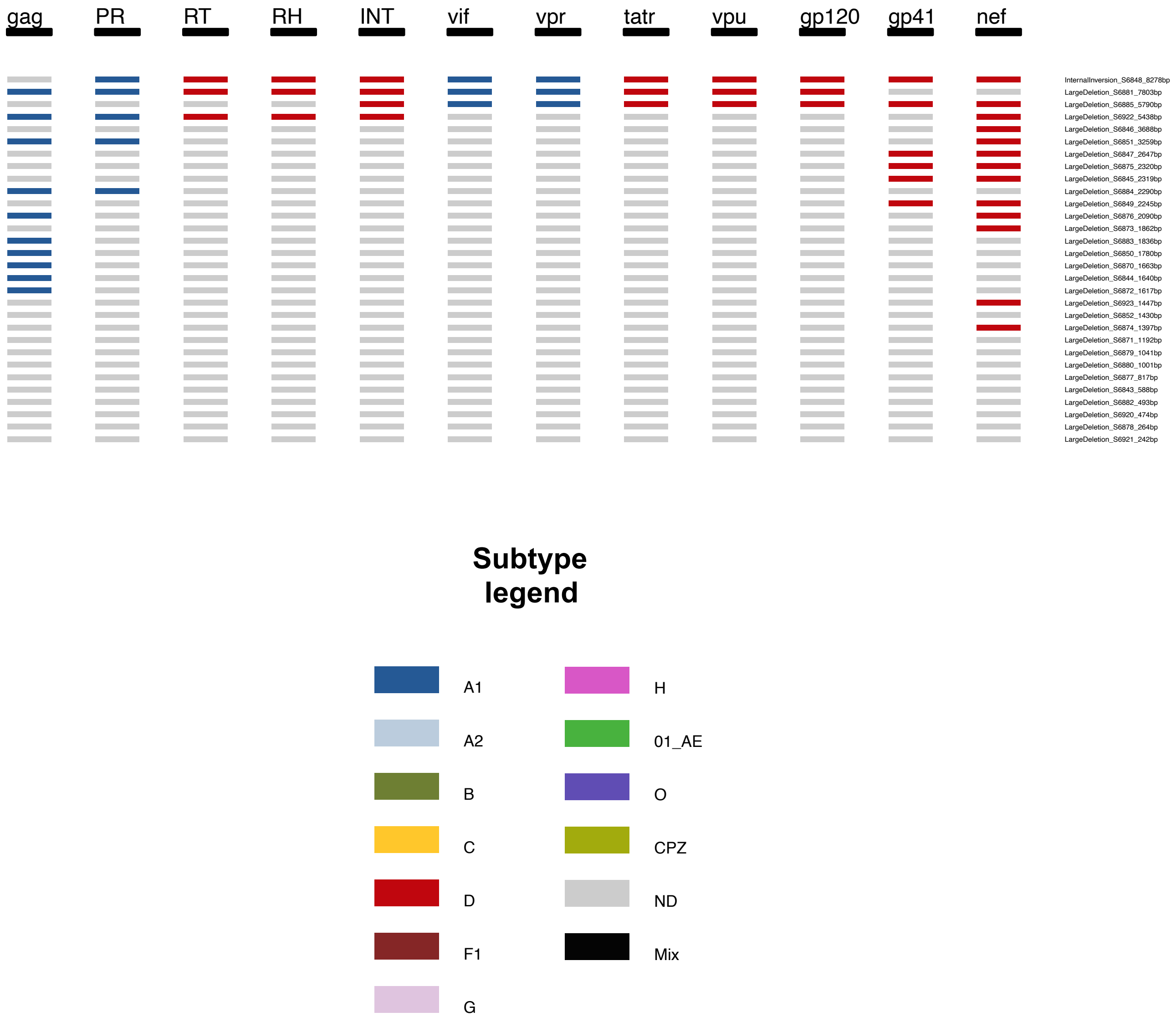

Figure S1: Full-genome HIV subtyping by FLIP-seq and MOCHI: Donor 18. Donor 18's viral subtype was designated as an A1/D recombinant, which features a complex mosaic of A1 and D regions.

Donor 19 – subtype A1/C/D

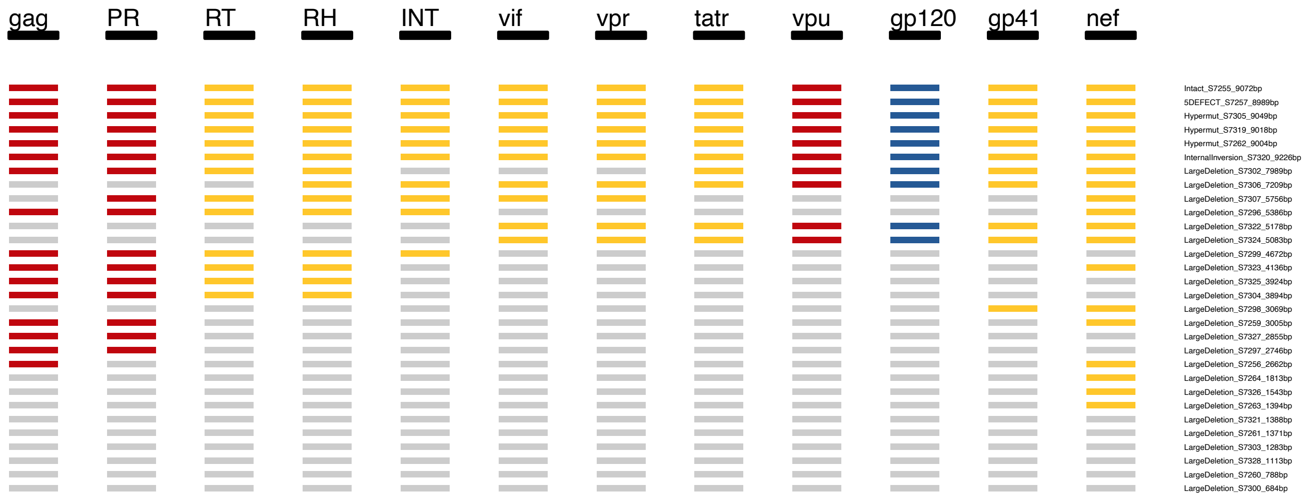

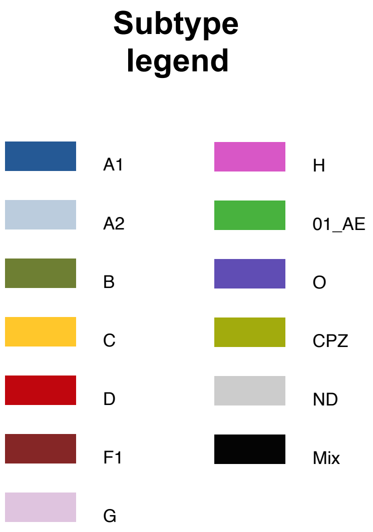

Figure S1: Full-genome HIV subtyping by FLIP-seq and MOCHI: Donor 19. Donor 19's viral subtype was designated as an A1/C/D recombinant.

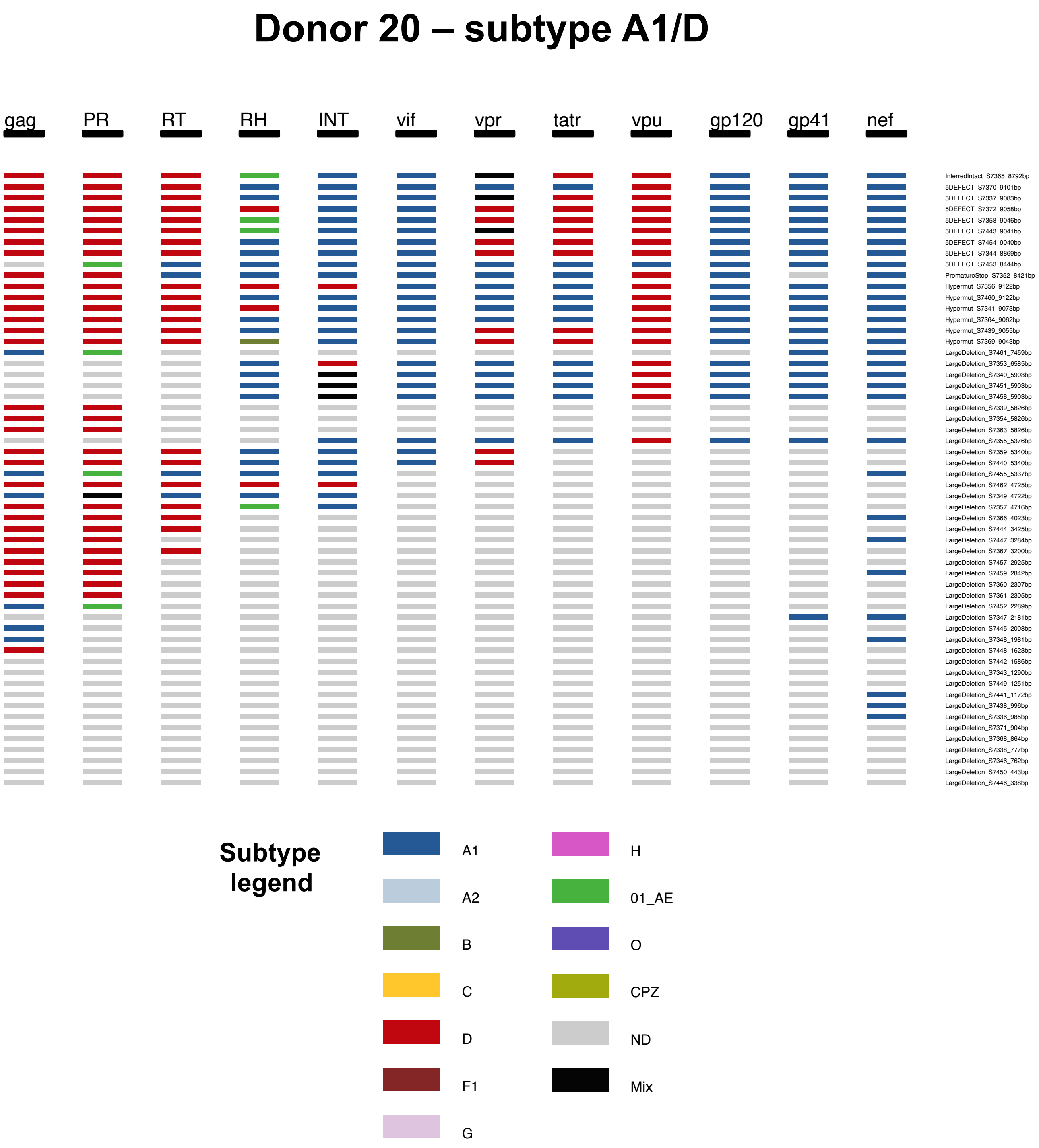

Figure S1: Full-genome HIV subtyping by FLIP-seq and MOCHI: Donor 20.
The subtype 01_AE calls observed in a minority of protease and RNAseH (RH) domain sequences were interpreted as A1, as 01_AE is subtype A1 within these 5' genomic regions. Similarly, the subtype B call within the RNAseH domain for one virus sequence was interpreted as likely subtype D, given the close evolutionary relatedness of these subtypes and the rarity of subtype B in Uganda. Donor 20's viral subtype was thus designated as an A1/D recombinant.

Donor 21 – subtype A1/D

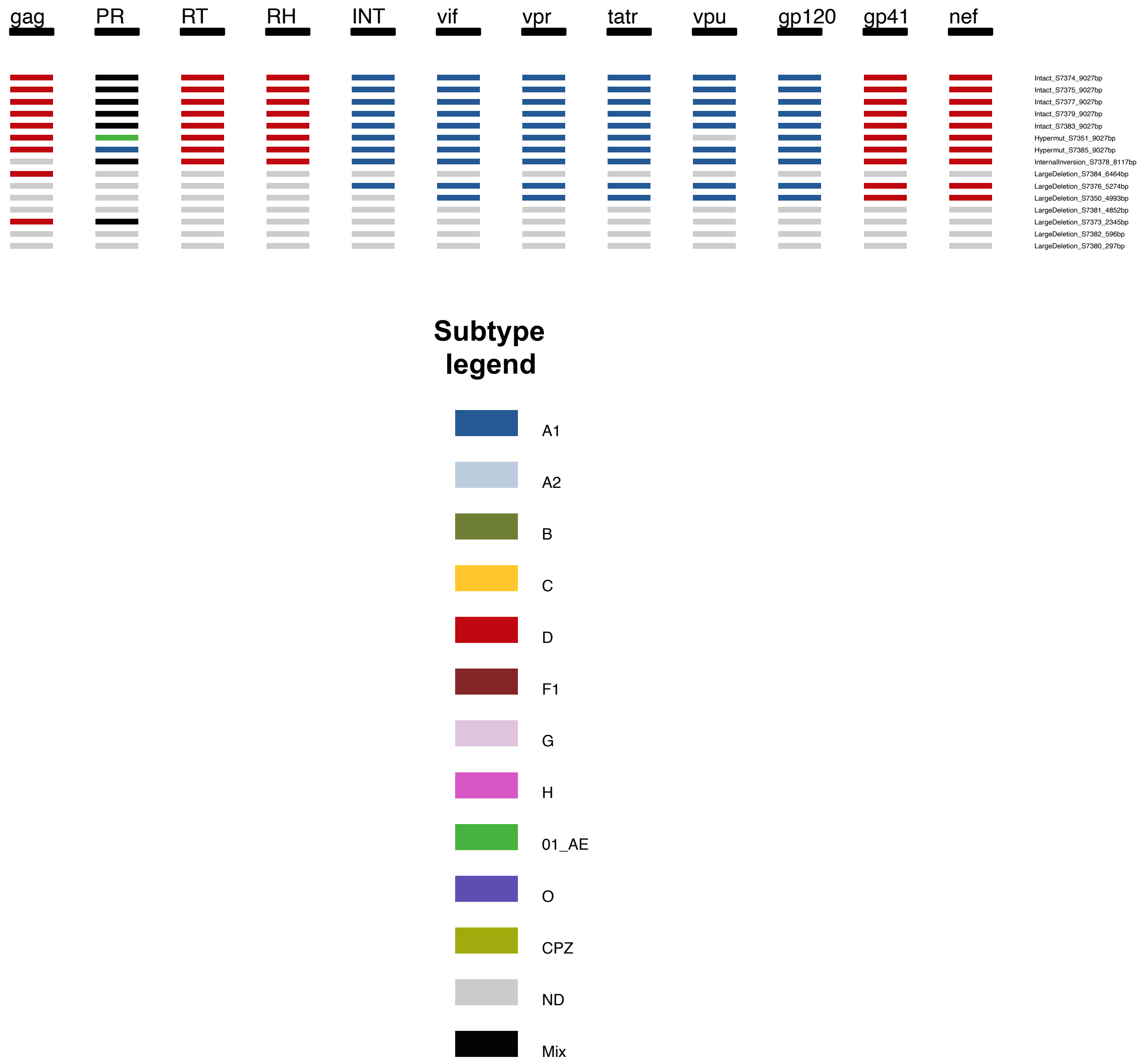

Figure S1: Full-genome HIV subtyping by FLIP-seq and MOCHI: Donor 21. The single subtype 01_AE call within protease was interpreted as A1, as 01_AE is subtype A1 within these genomic regions. Donor 21's viral subtype was designated as an A1/D recombinant.

Donor 22 – subtype A1

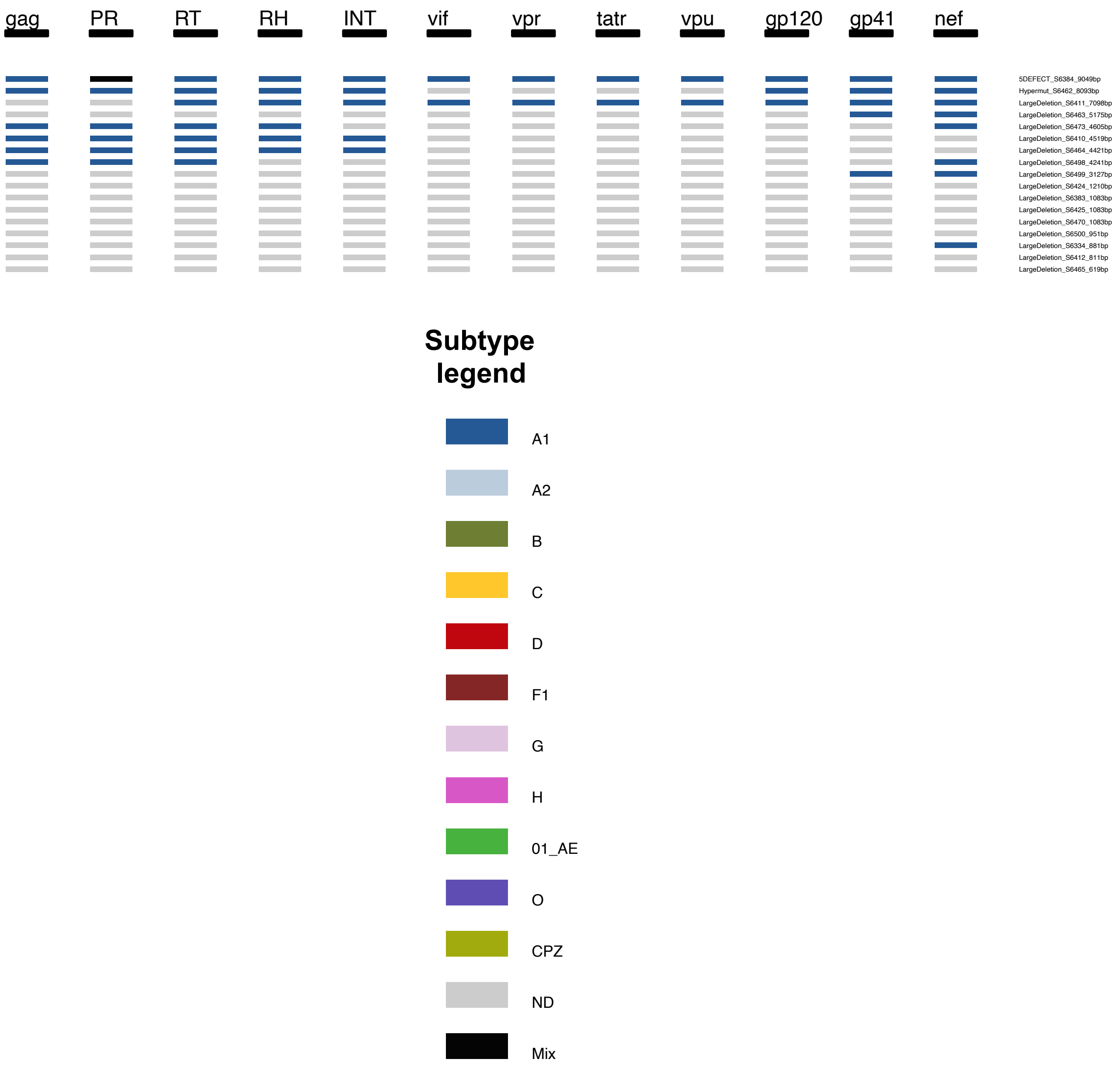

Figure S1: Full-genome HIV subtyping by FLIP-seq and MOCHI: Donor 22. Donor 22's viral subtype is A1.

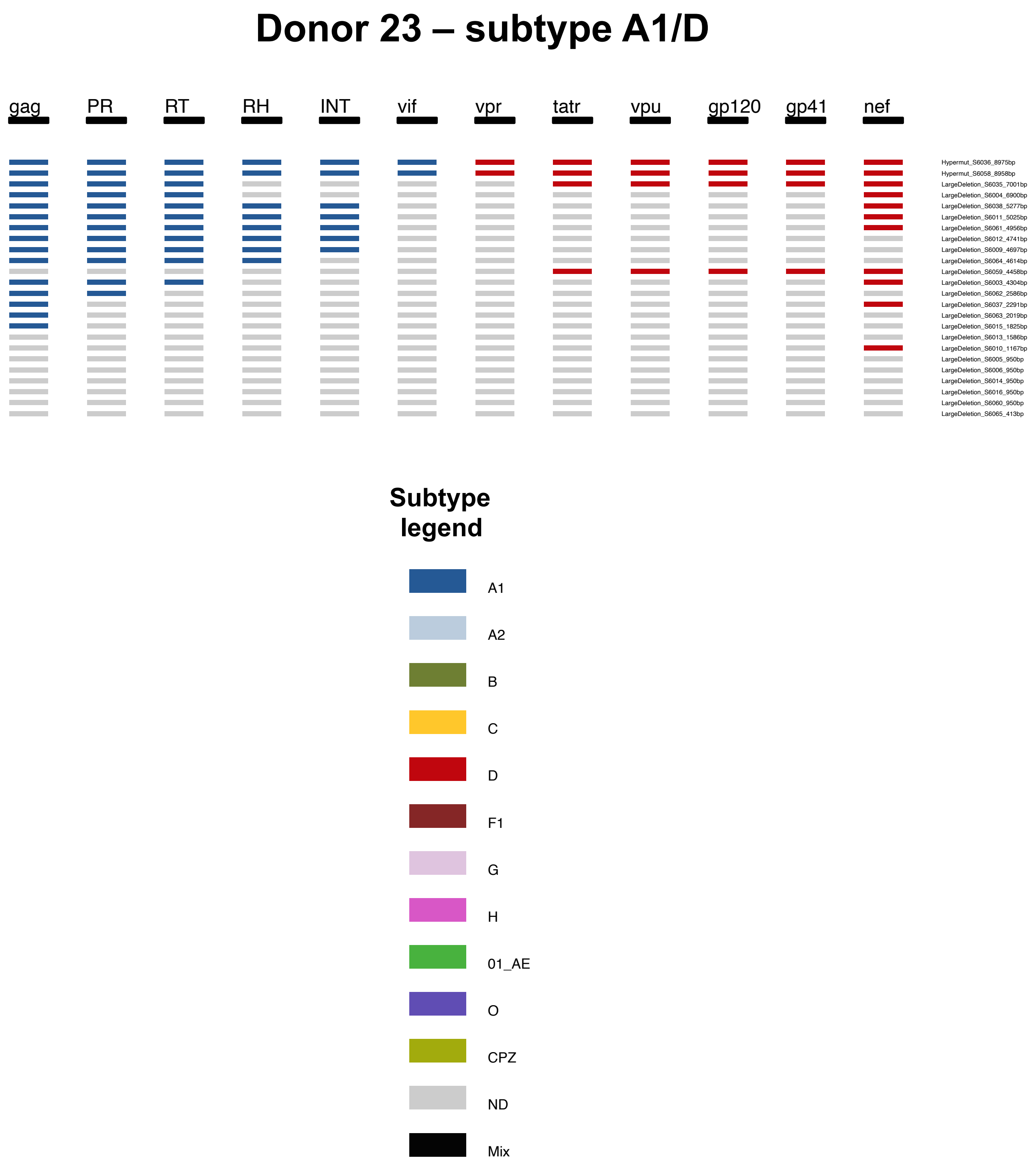

Figure S1: Full-genome HIV subtyping by FLIP-seq and MOCHI: Donor 23. Donor 23’s viral subtype was designated as A1/D recombinant, where the transition between A1 and D genomic regions occurs somewhere near the end of *vif* and the beginning of *vpr.*

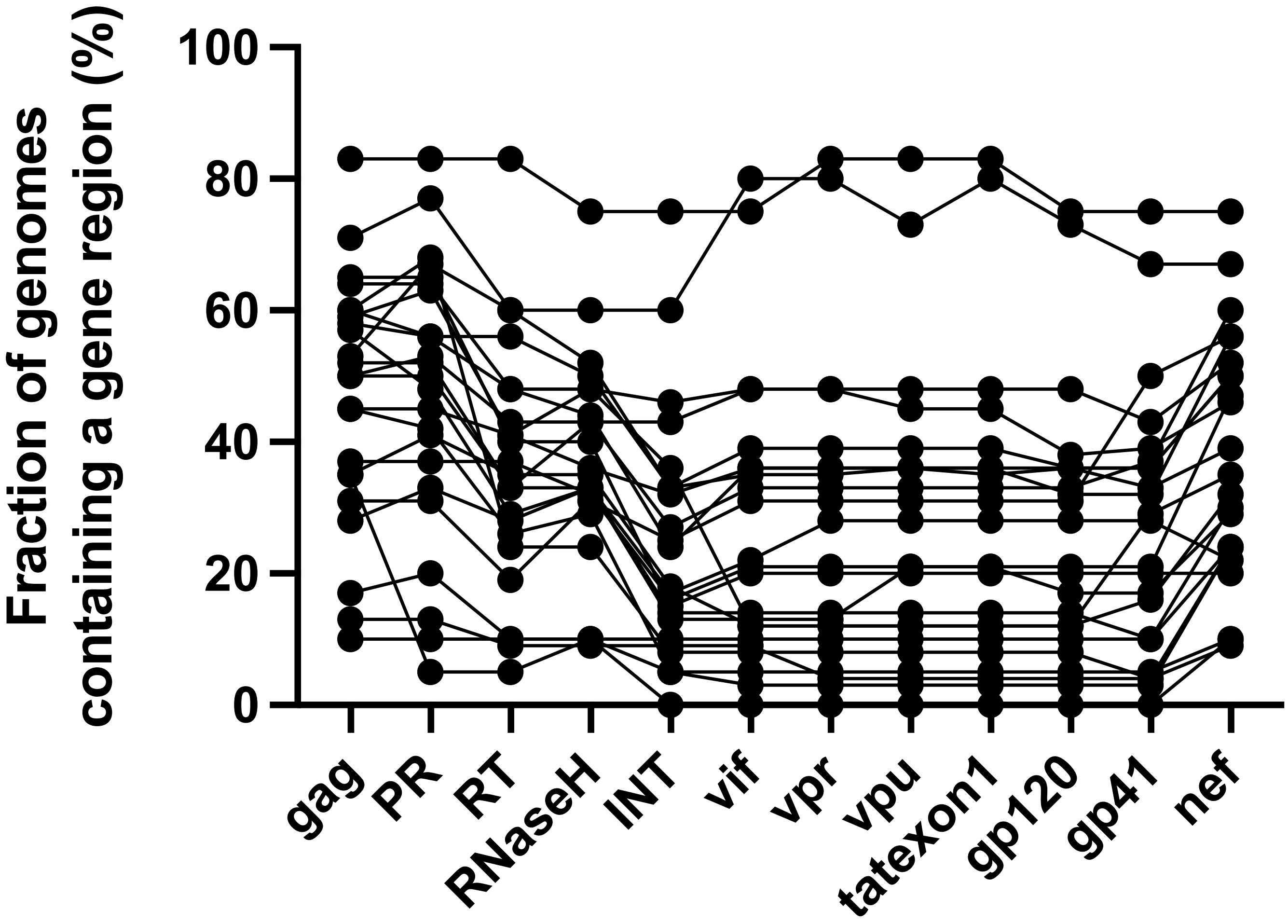

Figure S2. Commonly deleted HIV regions in RHSP cohort proviruses. Each dot represents the fraction of HIV-1 DNA genomes sampled from one individual that contained the specified genomic region. In this cohort of HIV-1 subtype A1, D and recombinant infections, genome truncations were most frequently observed in the region between RT and *env*. Relative to *gag*, p-values from the Wilcoxon signed rank test with continuity correction for these regions ranged between $p=4x10^{-5}$ and 0.0005. Compared to *gag*, median differences for these genes were 15% (RT), 15% (RNAseH), 32% (INT), 29% (*vif*, *vpr*, *vpu*, *tatexon1* and gp120) and 22% (gp41). Corresponding W-statistics were 166 (RT), 202 (RNAseH), 273 (INT), 239 (*vif*), 198 (*vpr*), 200 (*vpu*), 198 (*tatexon1*), 222 (gp120), 233 (gp41).

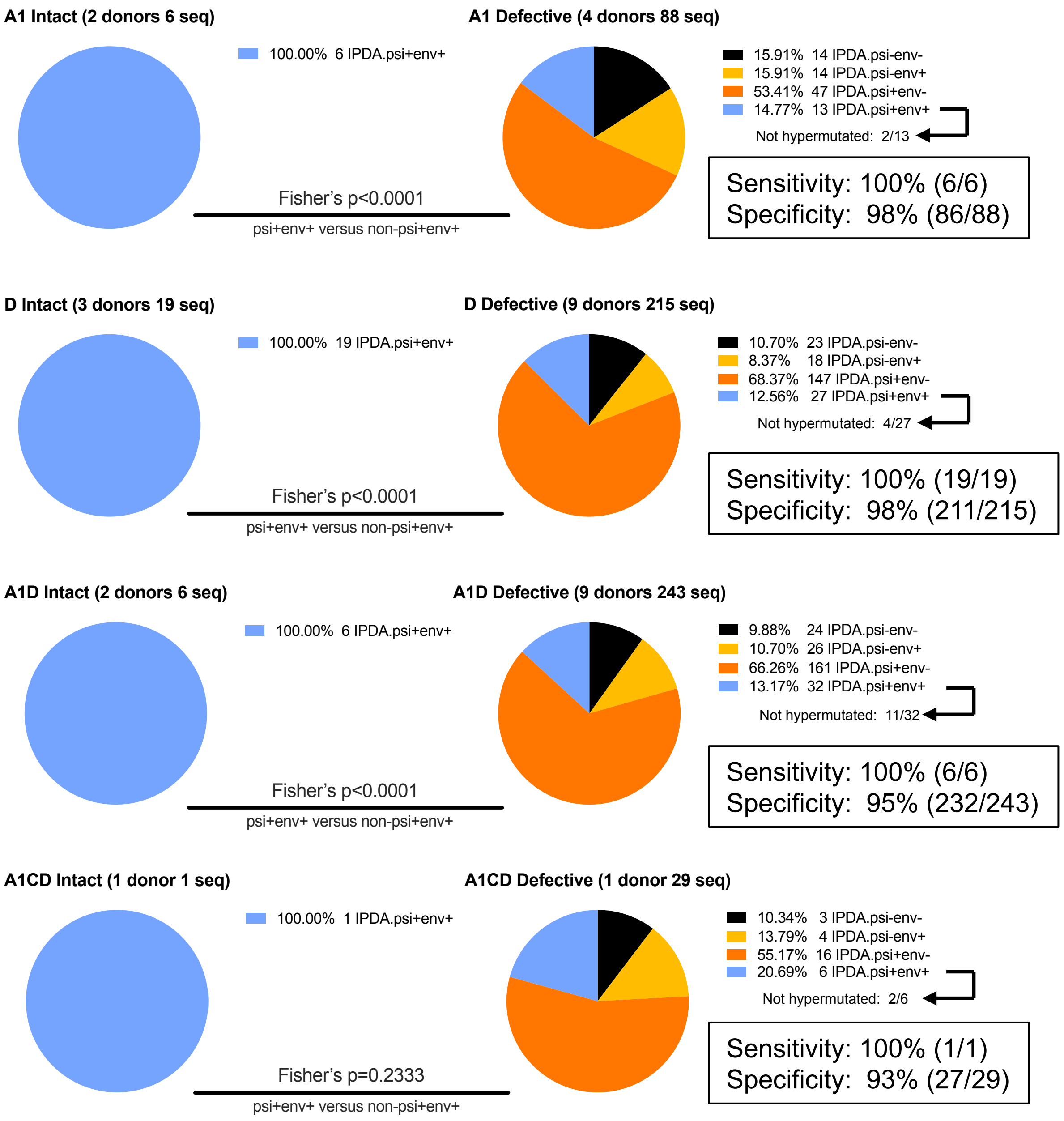

Figure S3. Inferred viral intactness as determined by IPDA *psi* and *env* primer and probe binding locations for subtypes A1, D and recombinants. Defective proviral sequences that contain intact *psi* and *env* regions (*psi*+*env*+) were further described for each subtype with the majority of these being hypermutated sequences. Odds ratios for all Fisher's exact tests returned values of infinity because, as expected, 100% of all intact sequences were "psi+env+".

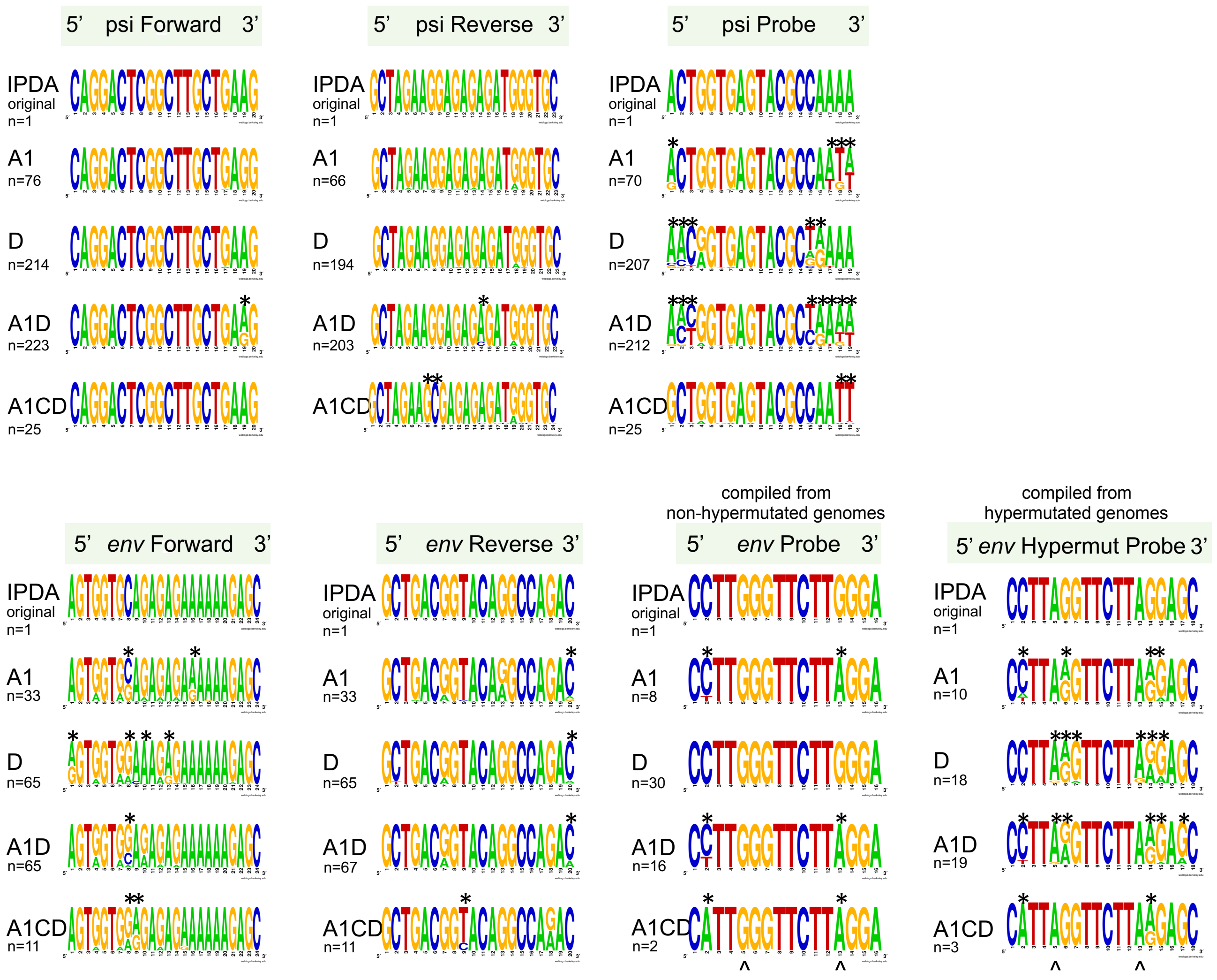

Figure S4. **Sequence diversity within the IPDA *psi* and *env* primer/probe regions in the RHSP cohort.** The top sequences are the published IPDA primer and probe region sequences, all shown in the forward direction (see Table S8 in this document for the actual primer/probe sequences). Mismatches are indicated with black asterisks (G>A mutations were excluded except in the labeling of *env* probes). Accent marks ^ at the bottom of the two *env* probes indicate the two positions in the original IPDA that were designed to discriminate hypermutated sequences.

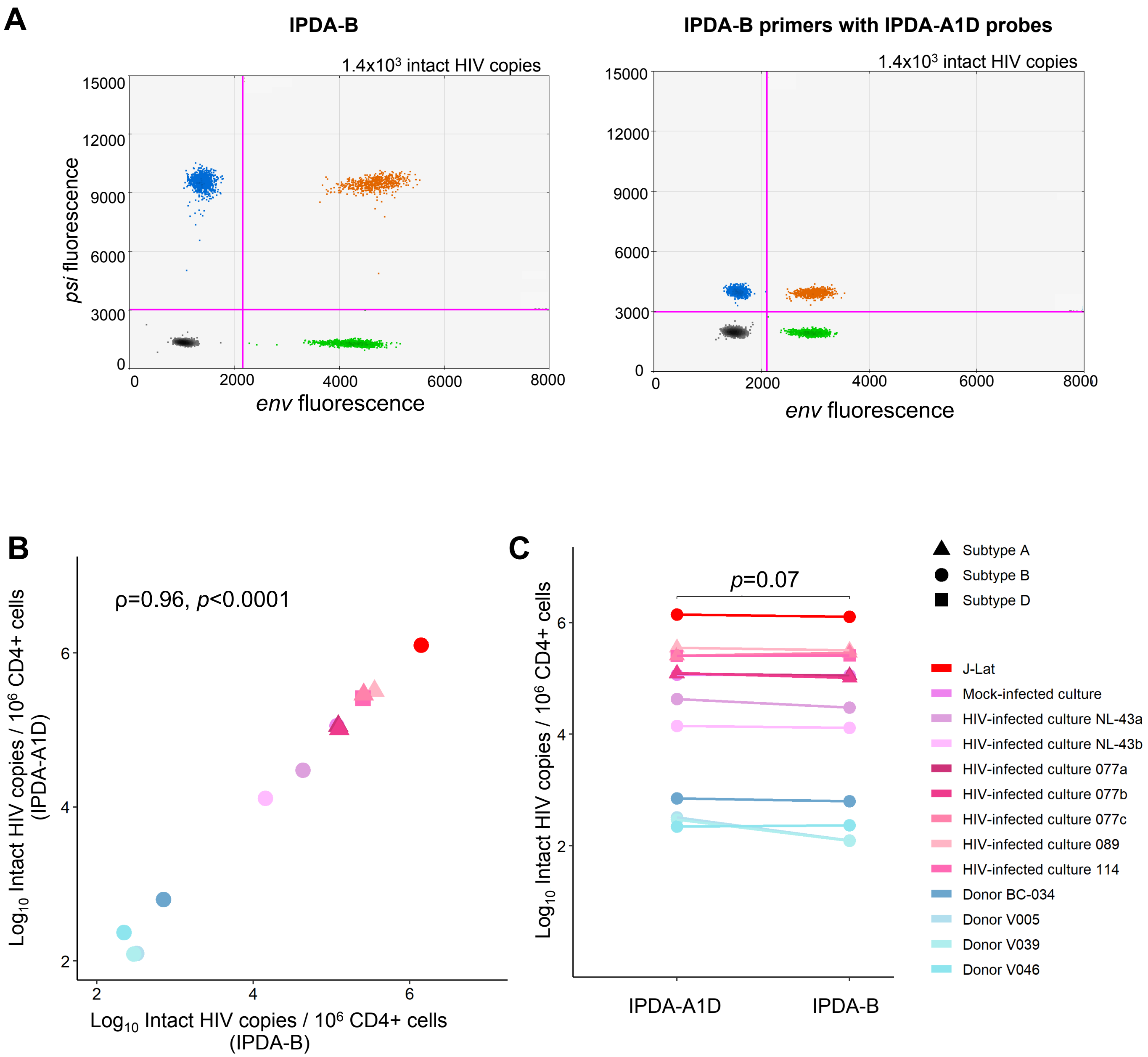

Figure S5. Comparison of IPDA-B IPDA-A1D. (**A**) IPDA-B (left) and a modified assay featuring IPDA-B primers and IPDA-A1D degenerate probes (right) applied to an HIV subtype B reference sequence. Though the IPDA-A1D probes reduced *psi* and *env* fluorescence amplitude, populations remained well-defined. (**B**) Spearman's correlation between IPDA-B and IPDA-A1D results for 13 samples with sequences compatible with both assays. Spearman's ρ=0.96 (95% Confidence interval 0.87-0.99); degrees of freedom=11; *p*<0.0001) (**C**) A Wilcoxon paired test applied to the raw (untransformed) data in (B) yielded a W-statistic (sum of signed ranks) of -53, a median [Interquartile range (IQR)] 117,094 [517-254,578] intact HIV copies/million CD4+ T-cells for IPDA-A1D versus a median 102,726 [IQR 432-273,114] intact HIV copies/million CD4+ T-cells for IPDA-B; with p=0.07. This indicates that there s no significant bias towards higher (or lower) values between assays. Further supporting this, Lin's concordance correlation coefficient ρ estimate was 0.9941 (95% Confidence interval 0.9885-0.9970), suggesting an almost perfect concordance between IPDA-B and IPDA-A1D.

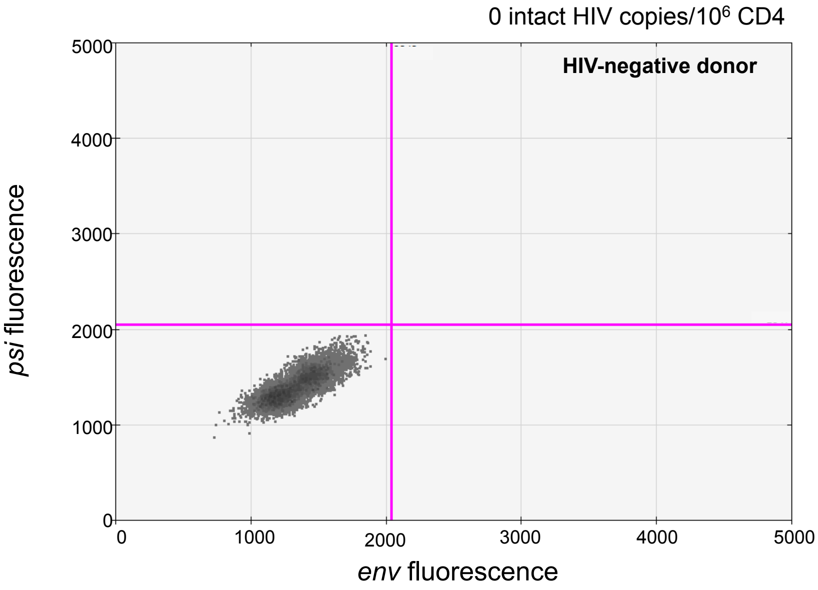

Figure S6. IPDA-A1D applied to a donor without HIV (negative control).

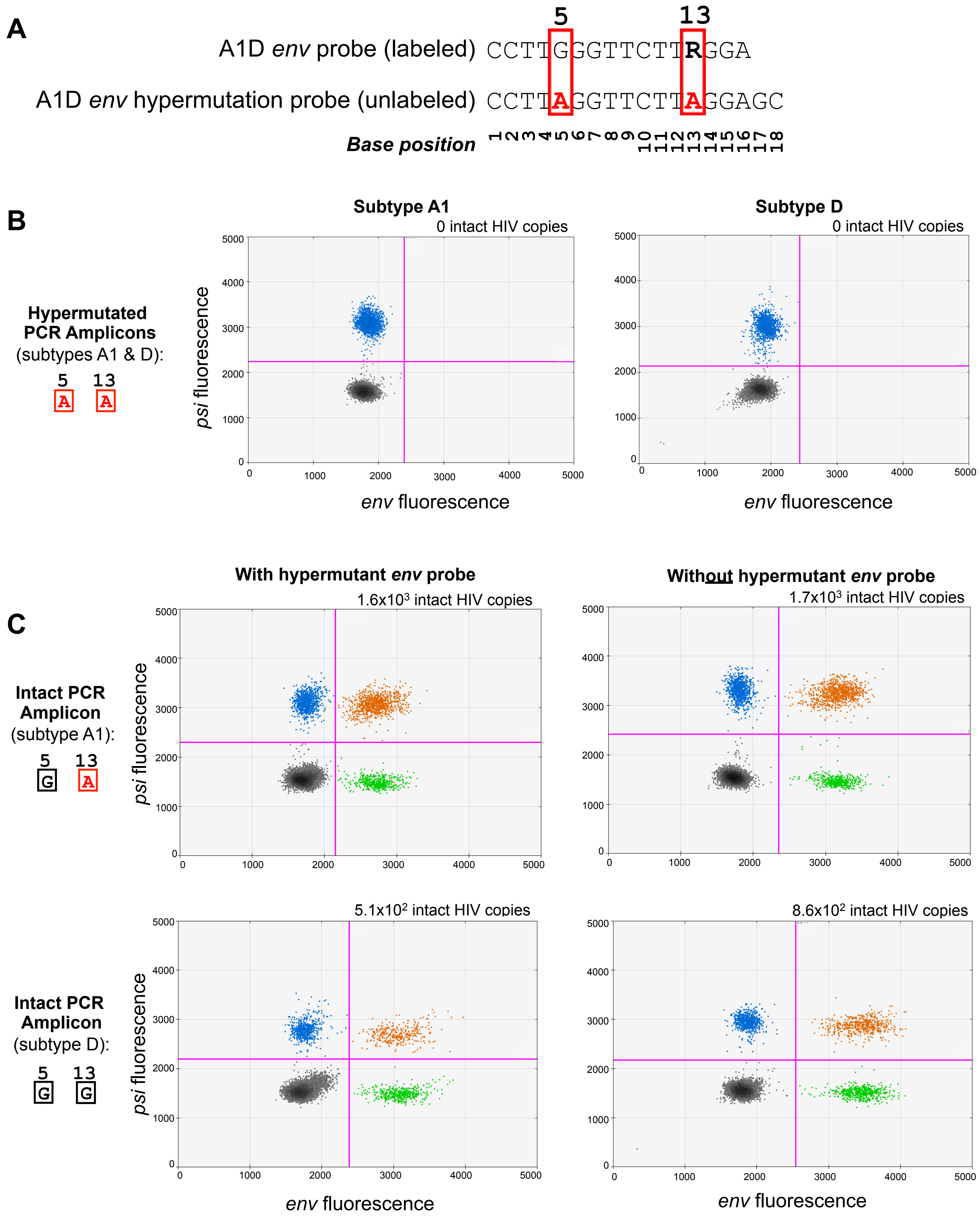

Figure S7. IPDA-A1D hypermutation discrimination validation. (**A**) IPDA-A1D *env* and hypermutation probe sequences, with hypermutation-discrimination bases 5 and 13 highlighted. (**B**) IPDA-A1D applied to a hypermutated amplicon from a subtype A1 donor (left) and a subtype D donor (right). Both had "A" bases at positions 5 and 13. (**C**) IPDA-A1D assay performed with the hypermutation probe (left panels) and without it (right panels), applied to an intact subtype A1 amplicon (upper panels) and an intact subtype D amplicon (lower panels). Their bases at positions 5 and 13 are shown at the far left.

Examination of the resilience of *IPDA-B* probes to the type of within-host HIV diversity seen in Donor 20

A   ***psi* probe region**

**IPDA-B**	`ACTGGTGAGTACGCCAAAA`
**IPDA-A1D**	`-MY-----------NR-W-`
**Donor 20 variant 1**	`-----------------TT`
**Donor 20 variant 2**	`-----------------G-`

B   ***env* probe region**

**IPDA-B**	`CCTTGGGTTCTTGGGA`
**IPDA-A1D**	`------------R---`
**Donor 20 variant 1**	`-T----------A---`
**Donor 20 variant 2**	`------------A---`

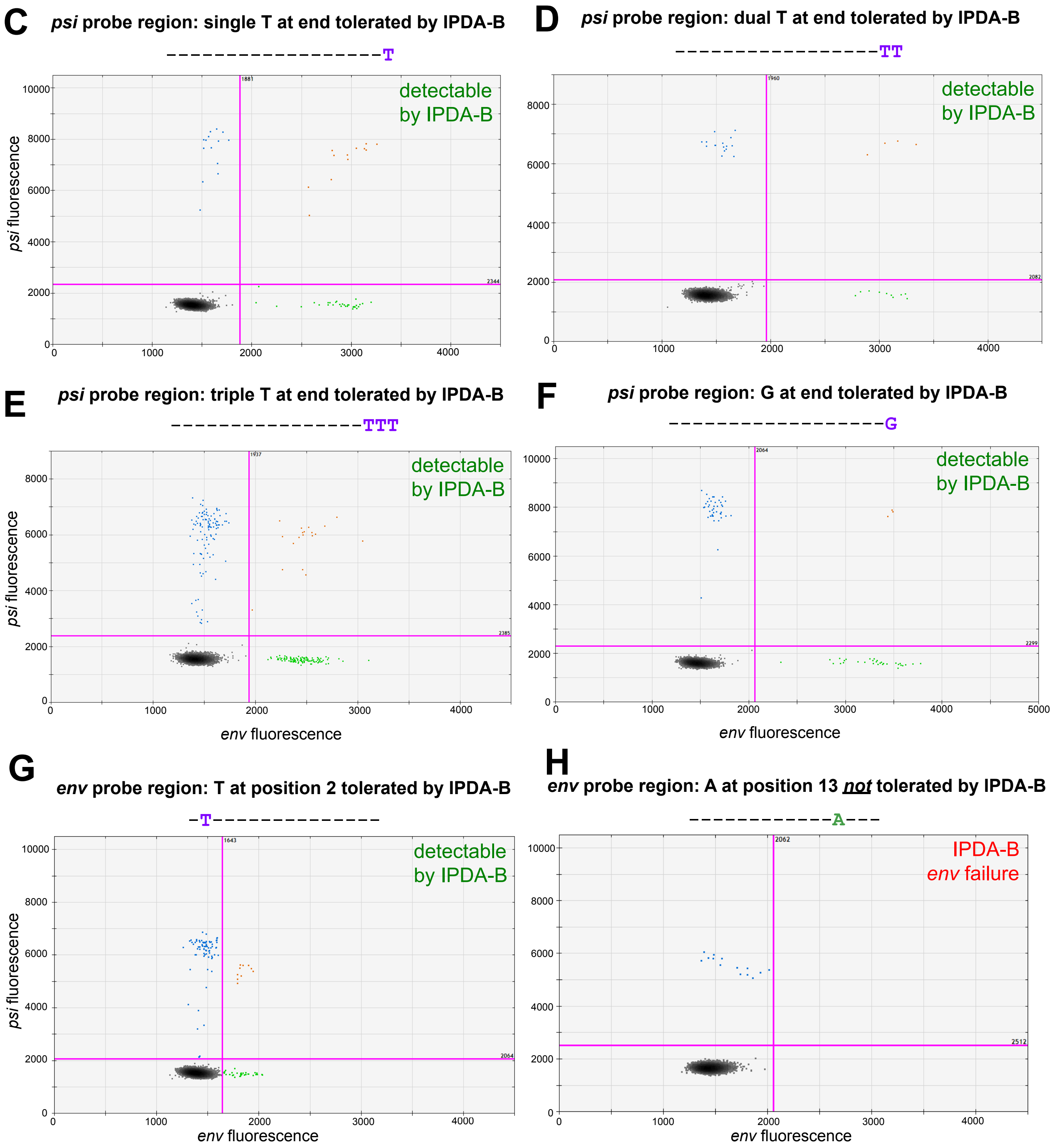

Figure S8 (previous page). Examination of the resilience of *IPDA-B* probes to the type of within-host HIV diversity seen in Donor 20.

Panels A and *B* show donor 20's co-dominant variant sequences in the *psi* and *env* probe regions, respectively. Dashes indicate a match to both assays, green bases indicate a mismatch to the IPDA-B that is captured by IPDA-A1D, and purple bases indicate mismatches to both assays.

As shown in Figure 4C, Donor 20's proviral pool yielded an *env* failure with IPDA-B, where detection was rescued by IPDA-A1D. Here, we draw from our knowledge of which polymorphisms are (or are not) tolerated by IPDA-B, to deconstruct Donor 20's assay results.

Panels C, D, E, F: The IPDA-B is very tolerant of mismatches near the end of the *psi* probe region. Here, we show representative data from participants whose proviral pools have up to three consecutive "T" mismatches at the end of the *psi* probe region (*panels C, D, E*). All are well-tolerated with minimal impact on amplitude. Though we did not have any examples of a participant with a single G at the penultimate position, participants with a "G" mismatch at the very end are also well tolerated (*panel F*). Reasoning that polymorphisms tolerated by the IPDA-B will also be tolerated by IPDA-A1D, these data support the notion that both IPDA-B and IPDA-A1D detected the *psi* variation in Donor 20.

Panels G, H. The IPDA-B tolerates a single "T" mismatch at the second position of the *env* probe region, though with reduced amplitude (*panel G*). We thus infer that the IPDA-A1D will also tolerate this polymorphism. The IPDA-B however does not tolerate an "A" at the fourth position from the end of the *env* probe; this causes assay failure (*panel H*). As this "A" is found in both of Donor 20's variants, this likely explains why IPDA-B produced an *env* assay failure for this donor (shown in Figure 4C). By contrast, the IPDA-A1D assay design captures this "A” with the degenerate “R” base at this position, which is consistent with our observation that IPDA-A1D rescued detection in Donor 20 (Figure 4C).

Taken together, these observations are consistent with Donor 20's IPDA-B and IPDA-A1D results shown in Figure 4C, and additionally support the notion that IPDA-A1D is likely capable of detecting both of Donor 20's co-dominant proviral species.

Table S1. Donor characteristics

ID	Sex	Years on ART	CD4 (count/μl)	Pre-ART viral load (copies/ml)
Donor1	Female	11	1346	674258
Donor2	Male	12	451	845224
Donor3	Female	5	593	NA
Donor4	Male	7	429	NA
Donor5	Female	11	515	12543
Donor6	Female	5	578	NA
Donor7	Female	12	654	11037
Donor8	Female	6	968	NA
Donor9	Male	4	678	NA
Donor10	Male	12	439	59388
Donor11	Male	5	875	NA
Donor12	Female	8	984	10178
Donor13	Female	11	649	526353
Donor14	Female	9	829	84428
Donor15	Female	12	901	150303
Donor16	Female	7	598	NA
Donor17	Female	11	777	29348
Donor18	Female	6	883	NA
Donor19	Male	11	761	15363
Donor20	Female	10	875	3800
Donor21	Female	6	921	NA
Donor22	Female	9	963	12201
Donor23	Male	9	454	34254

NA: Not Available.
All donors had undetectable plasma viral loads.
100% (23/23) of the donors received NRTI/NNRTI as their first regimen.

Table S2. Reservoir measurements

ID	Infectious Units per Million (IUPM) resting CD4+ T-cells	Total HIV DNA load (copies LTRgag /million rCD4)	Cells sampled in sequencing PCR	Count of viral genomes obtained	Count of intact genomes obtained	% Intact	Subtype by FLIP-seq*
Donor1	0.148	735	239433	19	0	<5%	D
Donor2	0.048	2906	56357	16	0	<6%	A1D
Donor3	0.323	1786	67253	21	2	10%	A1
Donor4	1.170	5914	40826	21	1	5%	D
Donor5	0.509	644	119479	23	0	<4%	D
Donor6	0.534	1359	128972	25	1	4%	A1D
Donor7	1.421	669	284153	22	0	<4%	D
Donor8	0.148	2343	54263	20	0	<5%	A1D
Donor9	0.323	3342	33720	18	0	<5%	D
Donor10	0.048	182	77739	10	0	<9%	D
Donor11	0.048	129	191365	38	0	<2%	A1D
Donor12	0.071	1214	74870	20	0	<5%	A1
Donor13	0.423	1154	103898	25	0	<4%	A1D
Donor14	8.210	1694	135563	25	0	<4%	D
Donor15	0.919	578	240168	12	1	8%	D
Donor16	1.306	1895	158494	84	17	20%	D
Donor17	40.519	1315	192597	36	4	11%	A1
Donor18	2.259	1407	192656	30	0	<3%	A1D
Donor19	1.100	1188	77912	30	1	3%	A1CD
Donor20	2.629	1316	137843	56	0	<2%	A1D
Donor21	1.100	838	204794	15	5	33%	A1D
Donor22	0.323	2138	122244	17	0	<5%	A1
Donor23	1.100	2556	32798	24	0	<4%	A1D

*See Figure S1 for further subtyping details

Table S3. Detailed breakdown of HIV DNA genome defectiveness categories, count data

ID	*psi* Defect	Hyper-mutated	Internal Inversion	Large Deletion	Premature Stop Codon in *gag*/*pol*/*env*	Inferred Intact	Intact	Total
Donor1	0	1	0	17	1	0	0	19
Donor2	0	2	1	13	0	0	0	16
Donor3	0	3	0	15	1	0	2	21
Donor4	0	0	0	19	1	0	1	21
Donor5	0	1	0	22	0	0	0	23
Donor6	0	4	0	20	0	0	1	25
Donor7	0	7	0	15	0	0	0	22
Donor8	0	0	0	20	0	0	0	20
Donor9	2	0	0	16	0	0	0	18
Donor10	0	0	0	10	0	0	0	10
Donor11	0	1	0	37	0	0	0	38
Donor12	0	1	0	19	0	0	0	20
Donor13	0	2	0	23	0	0	0	25
Donor14	0	0	1	24	0	0	0	25
Donor15	0	7	0	4	0	0	1	12
Donor16	6	2	1	57	0	1	17	84
Donor17	0	5	0	27	0	0	4	36
Donor18	0	0	1	29	0	0	0	30
Donor19	1	3	1	24	0	0	1	30
Donor20	8	6	0	40	1	1	0	56
Donor21	0	2	1	7	0	0	5	15
Donor22	1	1	0	15	0	0	0	17
Donor23	0	2	0	22	0	0	0	24

Table S4. Genome categories by subtype, count data

	A1 (4 donors)	D (9 donors)	A1/D recombinants (9 donors)	A1CD (1 donor)	Total
5DEFECT	1	8	8	1	18
Hypermut	10	18	19	3	50
InternalInversion	0	2	3	1	6
LargeDeletion	76	184	211	24	495
PrematureStop	1	2	1	0	4
Inferred Intact	0	1	1	0	2
Intact	6	19	6	1	32
Total	94	234	249	30	607

A 3x7 Fisher's Exact test on the first 3 columns of data yielded an adjusted Cramer's V of 0.02 (95% confidence interval 0.00 - 1.00); p=0.3. This test was two-sided.

A1CD recombinants were excluded from this calculation due to n=1 donor.

Table S5. Detailed breakdown of clonal sequences, count data

ID	Intact clones	Defective clones	Non-clonal	Total
Donor1	0	6	13	19
Donor2	0	7	9	16
Donor3	0	0	21	21
Donor4	0	0	21	21
Donor5	0	16	7	23
Donor6	0	0	25	25
Donor7	0	5	17	22
Donor8	0	3	17	20
Donor9	0	0	18	18
Donor10	0	3	7	10
Donor11	0	8	30	38
Donor12	0	0	20	20
Donor13	0	8	17	25
Donor14	0	11	14	25
Donor15	0	0	12	12
Donor16	12	8	64	84
Donor17	0	2	34	36
Donor18	0	0	30	30
Donor19	0	0	30	30
Donor20	0	8	48	56
Donor21	2	0	13	15
Donor22	0	3	14	17
Donor23	0	5	19	24

Note. In cases where 0 intact and 0 defective clones were detected, “percent clonal sequences” in the manuscript was calculated by converting 0 to 0.9 as the lower limit of detection. For example, in donor 19, “percent clonal sequences” was <3% (less than 0.9/30x100%).

Table S6. Detailed breakdown of clonal sequences, by subtype

	A1 (4 donors)	D (9 donors)	A1/D recombinants (9 donors)
Clonal	5	61	41
Non-Clonal	89	173	208
Total seq	94	234	249
%Clonal	5%	26%	16%

A 3x2 Fisher's exact test of all three groups yielded an adjusted Cramer's V of 0.14 (95% Confidence interval 0.06 - 1.00), p=0.0002

A Fisher's Exact Test on A1 versus D yielded an Odds Ratio 0.16; p<0.0001

A Fisher's Exact Test on A1 vs A1D yielded an Odds Ratio 0.29; p=0.007

All above statistical tests were two-sided.

Table S7. Count of hypermutated versus non-hypermutated genomes among all the defective genomes that are IPDA *psi*+*env*+.

	Not hypermutated	Hypermutated
**A1**		
5DEFECT	1	
Hypermut		10
Large Deletion		1
Premature Stop	1	
**A1CD**		
5DEFECT	1	
Hypermut		3
Large Deletion	1	1
**A1D**		
5DEFECT	6	
Hypermut		19
Internal Inversion	1	
Large Deletion	3	2
Premature Stop	1	
**D**		
5DEFECT	1	
Hypermut		18
Internal Inversion		1
Large Deletion	1	4
Premature Stop	2	

Table S8. Original (IPDA-B) and modified (IPDA-A1D) primer and probe sequences.

Assay	Location (HXB2 coordinates)	Direction	Sequence	Fluorophore / Quencher
IPDA-B	*psi* forward primer (692-711)	Forward	CAGGACTCGGCTTGCTGAAG	
	psi probe (758-740)	Reverse	TTTTGGCGTACTCACCAGT	FAM / MGB-NFQ
	psi reverse primer (797-775)	Reverse	GCACCCATCTCTCTCCTTCTAGC	
	env forward primer (7736-7759)	Forward	AGTGGTGCAGAGAGAAAAAAGAGC	
	env probe (7781-7796)	Forward	CCTTGGGTTCTTGGGA	VIC / MGB-NFQ
	env hypermut probe (7781-7798)	Forward	CCTTAGGTTCTTAGGAGC	unlabeled / MGB-NFQ
	env reverse primer (7851-7832)	Reverse	GTCTGGCCTGTACCGTCAGC	
IPDA-A1D	*psi* forward primer (692-711)	Forward	CAGGACTCGGCTTGCTGA**R**G	
	psi probe (758-740)	Reverse	T**W**T**YN**GCGTACTCACC**RK**T	FAM / MGB-NFQ
	psi reverse primer (797-775)	Reverse	GCACC**Y**ATCTCTCTCCTTCTAGC	
	env forward primer (7736-7759)	Forward	AGTGGTG**S**A**R**AGAGAAAAAAGAGC	
	env probe (7781-7796)	Forward	CCTTGGGTTCTT**R**GGA	VIC / MGB-NFQ
	env hypermut probe (7781-7798)	Forward	CCTTAGGTTCTTAGGAGC	unlabeled / MGB-NFQ
	env reverse primer (7851-7832)	Reverse	GTCTGGC**Y**TGTACCGTCAGC	

MGB-NFQ: minor groove binder non-fluorescent quencher.

Table S9. Four donors failing IPDA-B were rescued by IPDA-A1D

Assay	Donor ID	Intact copies/million CD4+ T cells	Total HIV copies/million CD4+ T cells
IPDA-A1D	Donor 07	137	1513
	Donor 08	97	1477
	Donor 17	223	2651
	Donor 20	254	2779
IPDA-B	Donor 07	Failed	Failed
	Donor 08	Failed	Failed
	Donor 17	Failed	Failed
	Donor 20	Failed	Failed

Supplemental section:

ddPCR and FLIP-seq primer/probe validation

ddPCR and FLIP-seq primer/probe validation

PCR is sensitive to sequence variation. We thus examined if the ddPCR primers and probe, which were used solely for initial quantification of total HIV DNA to guide FLIP-seq dilutions, and FLIP-seq oligonucleotides used in HIV subtypes B and C, would also be appropriate for subtypes A1 and D, which predominate in Uganda.

The primer/probe sequences investigated are shown below.

Table S10. ddPCR (total HIV DNA) and FLIP-seq primers and probes

Primer	Assay	Direction	HXB2 coordinates	Length	Sequence 5'-3'
LTRgagF	ddPCR	Forward	684 → 700	17	TCTCGACGCAGGACTCG
LTRgagR	ddPCR	Reverse	810 → 793	18	TACTGACGCTCTCGCACC
LTRgagP	ddPCR	Probe (Reverse)	789 → 772	18	**/6-FAM/**CTCTCTCCT**/ZEN/**TCTAGCCTC**/31ABkFQ/***
U5-623F	FLIP-seq	Forward	623 → 649	27	AAATCTCTAGCAGTGGCGCCCGAACAG
U5-601R	FLIP-seq	Reverse	9686 → 9662	25	TGAGGGATCTCTAGTTACCAGAGTC
U5-638F	FLIP-seq	Forward	638 → 666	29	GCGCCCGAACAGGGACYTGAAARCGAAAG
U5-547R	FLIP-seq	Reverse	9632 → 9604	29	GCACTCAAGGCAAGCTTTATTGAGGCTTA
U5-769F^	FLIP-seq (alt)	Forward	769 → 793	25	GCGGAGGCTAGAAGGAGAGAGATGG

* /6-FAM/ is the 5' fluorophore, /ZEN/ is the internal quencher and /31ABkFQ/ is the Iowa Black FQ 3' quencher

^ This is a new alternate 2nd round FLIP-seq primer explored in the present study (see below).

We began by comparing ddPCR and FLIP-seq primer and probe sequences against HIV subtype reference alignments using QuickAlign.
https://www.hiv.lanl.gov/content/sequence/QUICK_ALIGNv2/QuickAlign.html

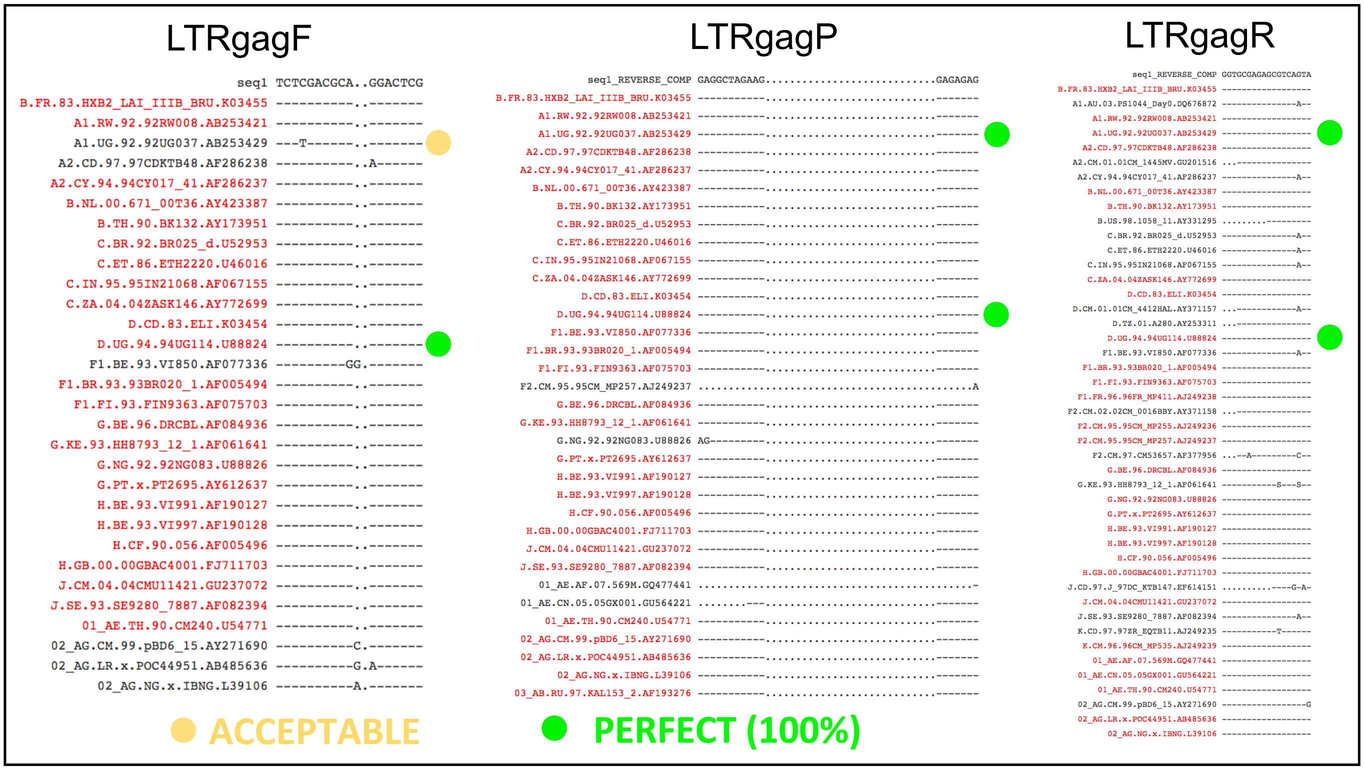

Figure S9. Comparison of ddPCR primers and probe to HIV subtype reference sequences (QuickAlign output). The indicated primer or probe region sequence is at the top, reverse-complemented if necessary. Below this are the HIV subtype reference sequences, where "-" characters indicate a match and "'." indicate gaps. Red sequence names indicate a perfect match to the reference sequence. The subtype reference sequences relevant to Rakai, A1 (A1.UG) and D (D.UG), are identified by coloured dots that specify the degree to which the primer or probe sequence matches the subtype reference.

In the present study, ddPCR was solely used to quantify total proviral load in order to calculate the dilutions needed to achieve single genome amplification (SGA) by FLIP-seq. Our analysis revealed that the ddPCR probe (LTRgagP) and reverse primer (LTRgagR) were 100% identical to the Ugandan subtype A1 reference, while the ddPCR forward primer (LTRgagF) had a single mismatch four bases from the 5' end that would not be expected to affect amplification (**Figure S9**). All three ddPCR oligos perfectly matched the Ugandan subtype D reference. We thus proceeded with the ddPCR primer/probe set without any modifications.

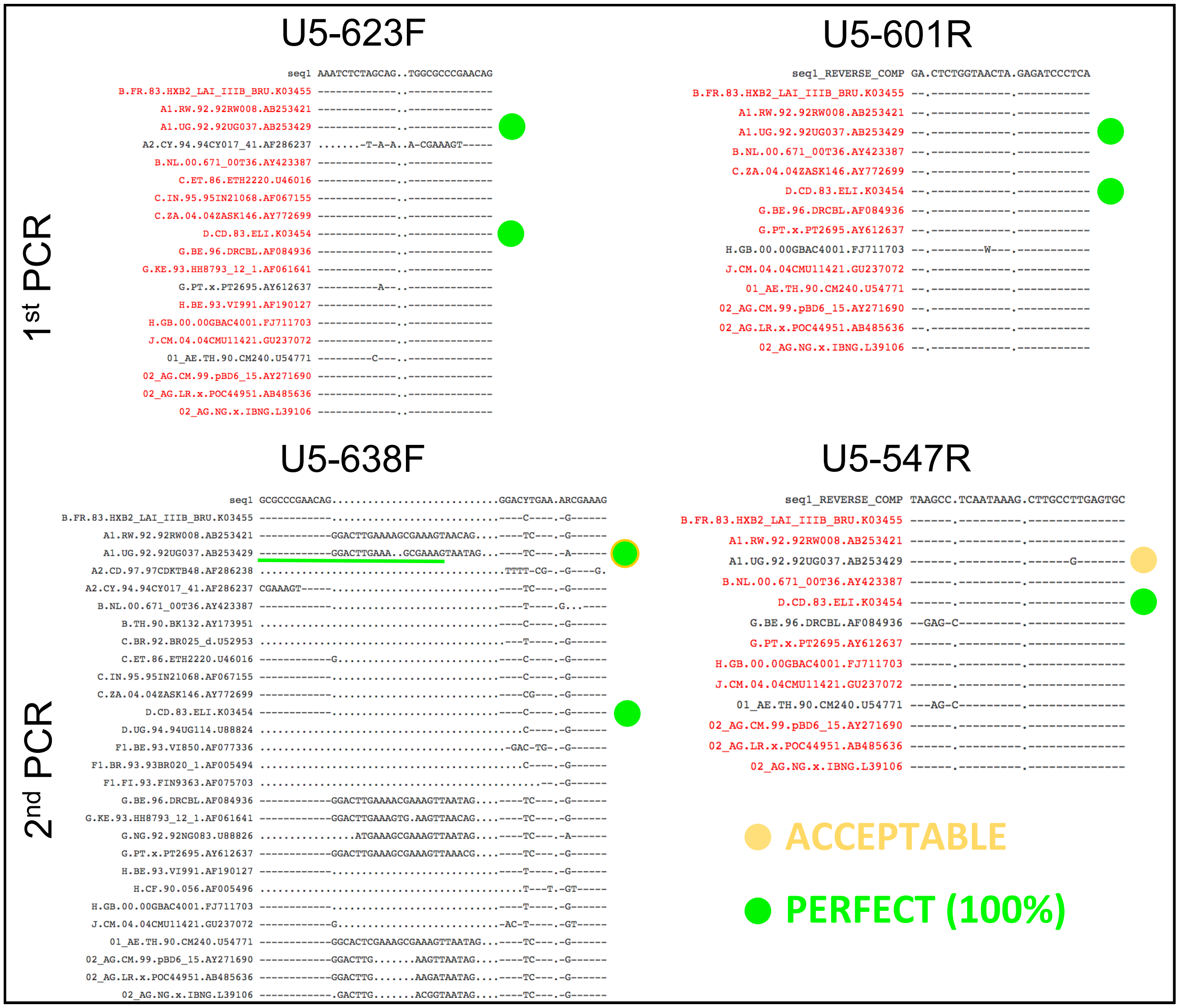

Figure S10. Comparison of FLIP-seq primers to HIV subtype reference sequences (QuickAlign output). The top sequence represents the indicated primer or probe region sequence, reverse-complemented if necessary. Below this are various HIV subtype reference sequences, where "-" characters indicate a match and "." indicate gaps. Red sequence names indicate a perfect match to the reference sequence. Relevant subtype A1 and D reference sequences are indicated by coloured dots that specify the degree to which the primer matches the subtype consensus. The A1.UG sequence has a large insertion in the U5-638F primer region, which nevertheless matches the primer sequence in the green underlined region. The dot adjacent to this sequence is therefore green with a yellow trim.

Both first-round PCR primers, U5-623F (forward) and U5-601R (reverse) shared 100% sequence identity with both subtype A1 and D reference sequences (here, a subtype D reference from the Democratic Republic of the Congo was used, as the Ugandan subtype D reference sequence did not have full coverage in this region) (**Figure S10**).

For the second-round forward PCR primer (U5-638F), the Ugandan subtype A1 reference sequence had a large insertion within this region, but the primer was nevertheless a perfect match for the first part of this repeated region (underlined in green). The second-round reverse primer (U5-547R) had a single base mismatch 8 bases from the 5' end that would not be expected to affect amplification. Both forward and reverse second round primers were a perfect match to the subtype D reference sequence.

We therefore anticipated that the FLIP-seq primers would be appropriate for the Rakai cohort. Nevertheless, given the insertions within U5-638F observed in the subtype A1 reference sequence, we designed an alternative second round forward primer, U5-769F (**Table S10**) that matched both the subtype A1 and D reference sequences perfectly (QuickAlign alignments not shown).

We then tested four second-round primer/probe combinations against one subtype A1 and one subtype D sample on a single 96-well plate with a total input of 30 copies of HIV DNA templates as quantified by ddPCR, as follows (all first round reactions used U5-623F and U5-601R):

Subtype A1

1. Original FLIP-seq (U5-638F and U5-547R) – yielded 11 genomes
2. LTRgagF and U5-547R – yielded 0 genomes
3. U5-623F and U5-547R – yielded 1 genome
4. U5-769F and U5-547R – yielded 12 genomes

Subtype D

1. Original FLIP-seq (U5-638F and U5-547R) – yielded four genomes
2. LTRgagF and U5-547R – yielded 0 genomes
3. U5-623F and U5-547R – yielded 0 genomes
4. U5-769F and U5-547R – yielded four genomes

These experiments suggested that only U5-769F had comparable efficiency to the original U5-638F forward primer.

Considering these observations, we proceeded with the original FLIP-seq first and second round primer set, as U5-638F captures the key 5' *psi* region of the viral genome.

Supplemental section:

Validation of HIVSeqinR for HIV subtypes A1 and D

Validation of HIVSeqinR for HIV subtypes A1 and D

HIVSeqinR is an R-based programming script that automates the bioinformatic inference of intactness of HIV-1 DNA genomes derived from the FLIP-seq protocol[1–3]. It has been extensively used in subtype B and C HIV-1 genetic studies[1,4–8]. In brief, HIVSeqinR identifies genomes containing large deletions of 1000 or more nucleotides, internal inversions, scrambled genomes, APOBEC 3G/3F-associated hypermutations, premature stop codons in *gag*/*pol*/*env*, and defects in the 5' *psi* region, and categorizes these as defective (**Figure S11**). All remaining genomes are putatively labelled genome-intact (**Figure S11**). When developing HIVSeqin R, we further validated the genome-intactness label by full-genome-sequencing outgrowth viruses from culture supernatants of positive wells derived from Quantitative Viral Outgrowth Assays (QVOAs), which by definition are genome-intact, to ensure HIVSeqinR would always label QVOA-derived viral genomes as "intact"[1]. The complete HIVSeqinR package is publicly available at https://github.com/guineverelee/HIVSeqinR.

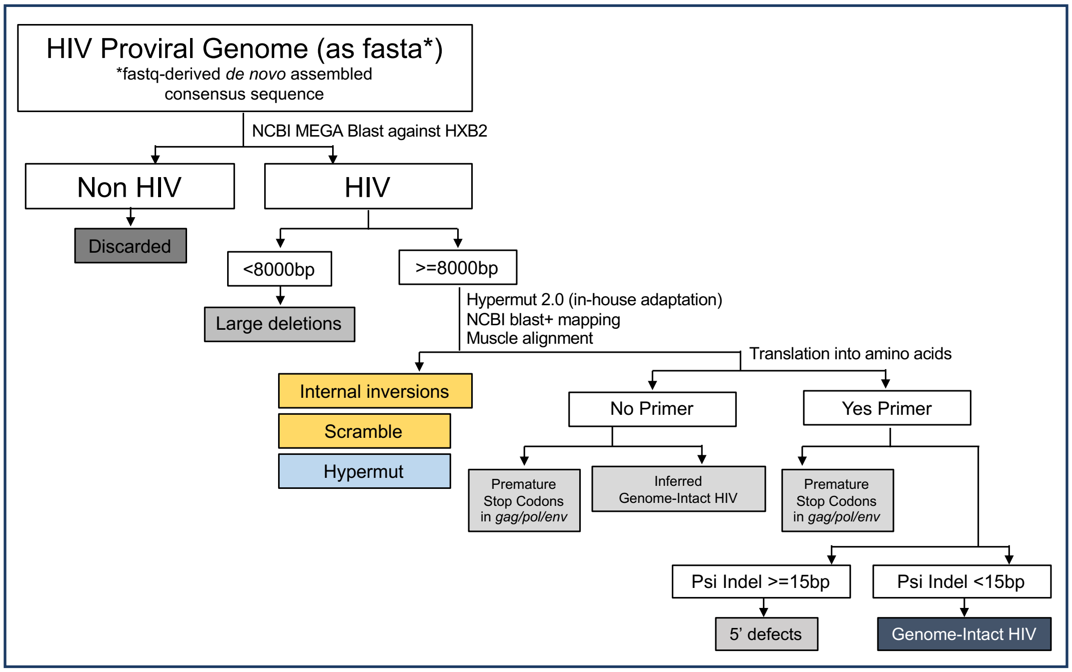

Figure S11. Published[1] HIVSeqinR decision tree for classification of viral genomes as defective versus intact

As HIV-1 displays extensive sequence diversity, we needed to ensure that HIVSeqinR correctly predicts genome-intactness in subtypes A1 and D. There are **four** main parameters in HIVSeqinR that could be sensitive to HIV subtype diversity, outlined below.

1. Choice of BLASTN reference sequence

HIVSeqinR takes as input *de novo* assembled contigs derived from FLIP-seq. As a first step in the workflow, NCBI BLASTN is used to map each contig against the HIV subtype

B reference sequence HXB2 to determine whether the sequence is HIV (TRUE HIV) or if it is a non-specific amplicon (FALSE HIV). We previously showed that, regardless of whether we used HXB2 or a subtype C reference sequence for this step, it did not change the TRUE/FALSE HIV determination of subtype C sequences[1]. Here, we tested the Ugandan subtype A1 reference sequence Ref.A1.UG.92.92UG037.AB253429 ("Ref.A1") and the Ugandan subtype D reference sequence Ref.D.UG.94.94UG114.U88824 ("Ref.D"), both retrieved from the Los Alamos HIV sequence database.

When we ran the 1022 assembled contigs from our study through BLASTN against reference sequences HXB2, Ref.A1 and Ref.D, we obtained the following:

HXB2 identified 721/1022 contigs as HIV.
Ref.A1 identified 731/1022 contigs as HIV (4% more compared to HXB2).
Ref.D identified 709/1022 contigs as HIV (2% fewer compared to HXB2).

Further investigation revealed that 100% of the contigs that yielded discordant HIV classifications were between 144-5055 nucleotides in length (**Figure S12**), with no discordances occurring among intact HIV calls. Given that: 1) these discordances only minorly impacted the overall HIV sequence counts due to small differences in the “large deletion” category, 2) no single reference will be 100% predictive for all subtypes, 3) multiple subtypes and intersubtype recombinants co-circulate in many regions including Uganda, and 4) bioinformatics automation should take priority over minimal gains achieved by modifying the reference sequence, we proceeded with using HXB2 as the reference sequence for the TRUE/FALSE HIV determination step.

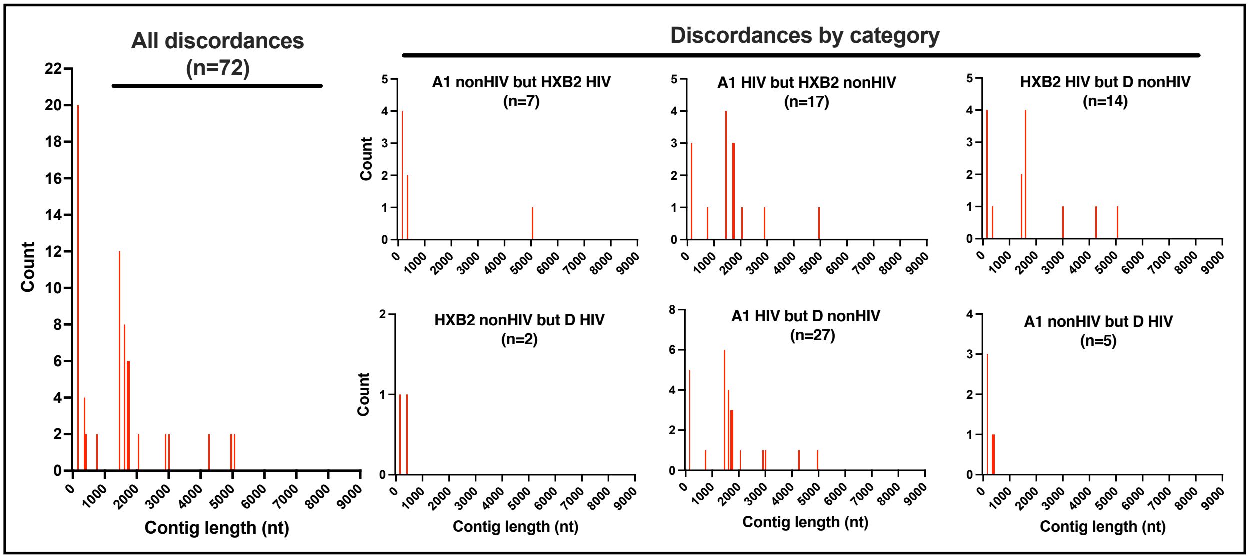

Figure S12. Lengths of discordantly classified contigs when different BLASTN reference sequences were used.

2. Choice of Hypermut reference sequence

HIVSeqinR contains an R-adaptation of the Los Alamos Hypermut 2.0 tool[9] with no changes to the algorithm. Briefly Hypermut 2.0 identifies APOBEC 3G/3F-associated G-to-A mutations in a query genome compared to a reference genome, and performs a Fisher's Exact test to evaluate whether G-to-A mutations in the query are enriched at known APOBEC motifs relative to non-motifs. Significance is assessed using a Fisher's exact test, and sequences with p<0.05 are deemed defective due to hypermutation. Again, the choice of reference sequence could impact this classification.

To test this, we multiple-aligned the 106 genomes collected in our study that lacked large deletions, internal inversions, or scrambled gene regions, and analyzed them for hypermutation using HXB2, Ref.A1 and Ref.D as Hypermut 2.0 reference sequences.

As expected, the p-values for each sequence differed marginally, confirming that Hypermut 2.0 does classify some G-to-A transitions differently based on which reference sequence is used. Despite this, the TRUE/FALSE hypermutation calls were 100% concordant regardless of which reference was used. Based on these results, and noting the bioinformatics advantages of keeping settings consistent in a given workflow, we retained HXB2 as the reference genome in the Hypermut 2.0 step. Note however that if one's study purpose is to specifically identify APOBEC-mediated transitions in a viral genome, not just globally classify it as hypermutated verus not, we underscore the Los Alamos HIV database's recommendation to use a donor- or subtype-specific reference sequence.

3. Definition of premature stop codons

HIVSeqinR also searches for premature stop codons in the three essential genes *gag*, *pol* and *env.* As we have previously discussed[2], "premature" is a relative term, as stop codons closer to the 5' end of a gene are highly likely to be detrimental while those closer to the 3' end might be tolerated. Recognizing that a number of HIV genes, including *gag* and *env*, commonly show length variation within and between-hosts, HIVSeqinR seeks to maximize specificity by allowing the lengths of *gag*, *pol* and *env* in a given proviral sequence to be 95-120% of those of the HIV reference strains HXB2 and NL4-3. Sequence lengths outside of this range will be deemed defective. We therefore needed to investigate whether these cut off values are appropriate for subtype A1 and D Gag/Pol/Env amino acid (AA) lengths.

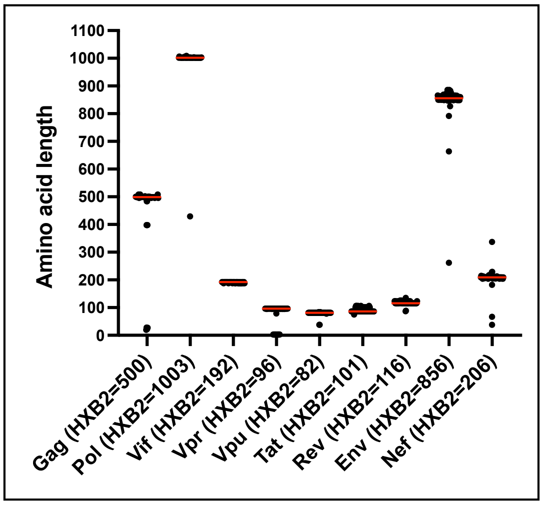

Figure S13. HIV protein lengths in the Rakai cohort. Red horizontal lines denote medians.

From the full dataset of 607, 56 remained after removing proviruses containing large deletions, hypermutations, internal inversions and scrambled genomic regions. When we ran

these 56 genomes through HIVSeqinR using the default settings, only four were flagged as having Gag, Pol or Env AA lengths outside of the acceptable range, where all were due to premature stop codons. This frequency was highly comparable to subtype B datasets[4,5]. We also visually inspected Gag/Pol/Env alignments to ensure there were no anomalies. Translation of all HIV genomic regions in these 56 sequences revealed that all viral proteins, particularly Gag/Pol/Env, had highly similar AA lengths to HXB2 and NL4-3 (below, and **Figure S13**):

Gag median AA length: 499 (HXB2/NL4-3 500)
Pol median AA length: 1002 (HXB2/NL4-3 1002)
Env median AA length: 856 (HXB2/NL4-3 856/854)

Together, these observations suggest that HIVSeqinR's 95-120% amino acid length cut-off is appropriate for subtypes A1 and D, so we retained it in our study.

4. Definition of 5' DEFECT

HIVSeqinR defines 5' DEFECT as "the presence of ≥15 nucleotide insertions or deletions in the 5' untranslated region of the genome between HXB2 nucleotide coordinates 638 to 789 (which extend from the start of the 2nd round PCR amplicon to the base before the start of *gag*)". This region includes the HIV packaging loop *psi* (ψ), a non-coding region that maps to HXB2 coordinates 681-789, and the 5' untranslated region upstream of *psi* (HXB2 638-680), which includes the Lys tRNA primer binding site. As previously discussed[2], there are currently no algorithms that can accurately predict the 3D RNA structure, nor genomic intactness, of a given *psi* sequence, nor of the 5' untranslated region upstream of *psi*. When HIVSeqinR was first developed, we decided to arbitrarily allow nucleotide length variation of up to 10% of that of HXB2 and NL4-3 in this region, hence the 15 nucleotide insertion/deletion cutoff (bases 638-789 span 152 nucleotides). Importantly, using this definition, HIVSeqinR did not incorrectly label any QVOA-derived subtype B viral sequence as "5' DEFECT" in our original validation[4]. However, little is known about the *psi* region in subtypes A1 and D, so we needed to validate this cut off value for these subtypes.

To better understand 5' untranslated region and *psi* sequence variation in replication-competent subtype A1 and D viruses, we sequenced viral RNA from p24-positive QVOA culture supernatants from 18 unique donors in the Rakai cohort. We only selected wells that were predicted to have originated from a single viral variant, as determined by Illumina sequencing of *pol* (HXB2 2723–3225) and *gp41* (HXB2 7938–8256) followed by analysis using a published Bayesian approach[10]. We also ensured that we sampled a variety of subtypes (4 putative A1; 9 putative D; 5 recombinants based on short-range Illumina sequencing of *pol* (HXB2 2723–3225) and *gp41* (HXB2 7938–8256), see Methods section of the main manuscript). These 18 QVOA culture supernatants were subjected to cDNA generation using oligo dT primers, followed by near-full-genome amplification using an overlapping five-amplicon approach developed for Ugandan samples that we have previously published[11]. The *psi* regions of these sequences are shown in the bottom half of **Figure S14**.

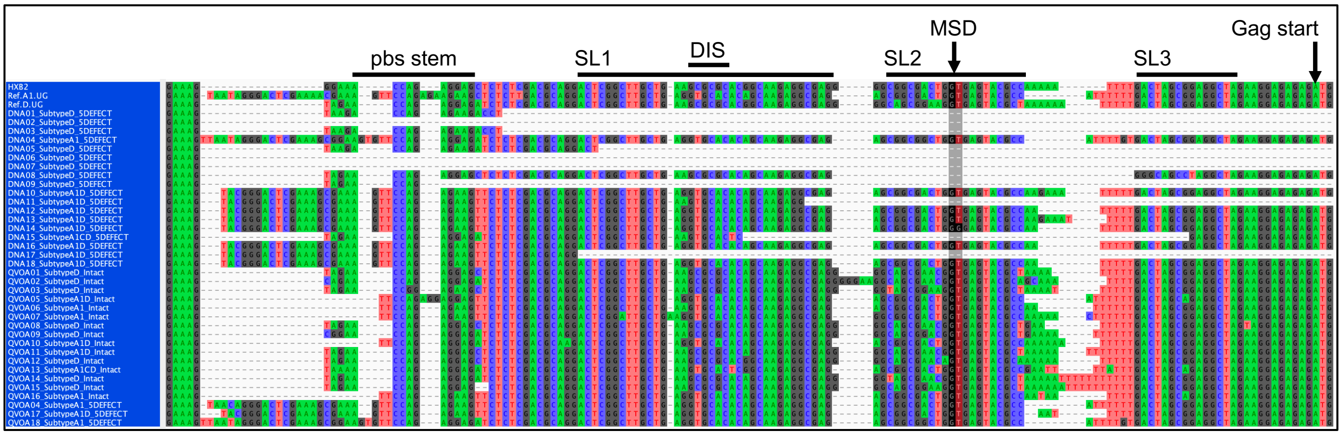

Figure S14. *psi* region alignments of 18 QVOA viral RNA genomes categorized as intact (bottom half) and 18 DNA genomes categorized as 5' defective (top half)

Of these 18 QVOA sequences, HIVSeqinR erroneously categorized three (QVOA04, QVOA17 and QVOA 18), as "5' defective" rather than the expected "intact" (**Figures S14 and S15**). This indicated that HIVSeqinR can predict genome intactness of subtype A1 and D sequences with an estimated 83% sensitivity.

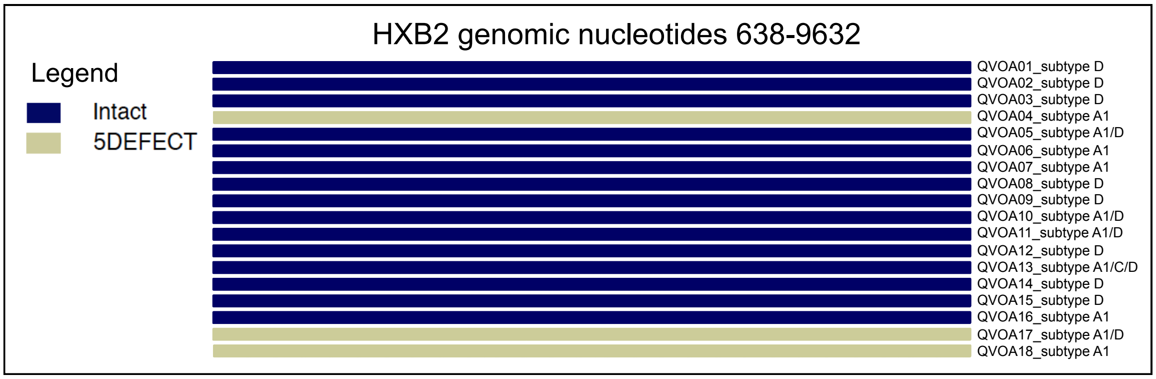

Figure S15. HIVSeqinR predicts subtype A1 and D genome intactness with 83% sensitivity

Of the 607 proviruses generated in the present study, HIVSeqinR classified 18 (3%) as having 5' defects (see sequences in the top half of **Figure S14**). Of these 18, 11 were obviously defective, as they harbored deletions of 44-121 nucleotides relative to HXB2/NL4-3 and were missing at least one of the SL1, SL2 or SL3 packaging loops (see sequences DNA01, 02, 03, 05, 06, 07, 08, 09, 11, 15 and 17; **Figure S14**). The remaining 7 had smaller insertions and/or deletions that were not represented in either of the subtype A1 or D reference sequences, and were thus less obvious to classify. Among these were 6 (DNA10, 12, 13, 14, 16, 18) that harbored a large insertion that was also observed in the QVOA 17 sequence (second-to-last sequence in **Figure S14**), but also harbored differences downstream between SL2 and SL3. In the absence of experimental evidence that these sequences can replicate, we retained them in the 5' defect category to maximize specificity. The final sequence, DNA04, was identical in

the *psi* region to QVOA 18 (last sequence in **Figure S14**), suggesting that its *psi* region is in fact functional.

Unfortunately, the 18 QVOA-derived viral RNA sequences each displayed substantial diversity in *psi* (**Figure S14**), and the three genomes classified as having 5' defects were not of the same subtype (two subtype A1, one subtype D1 at the 5' end of the genome, **Figure S16**).

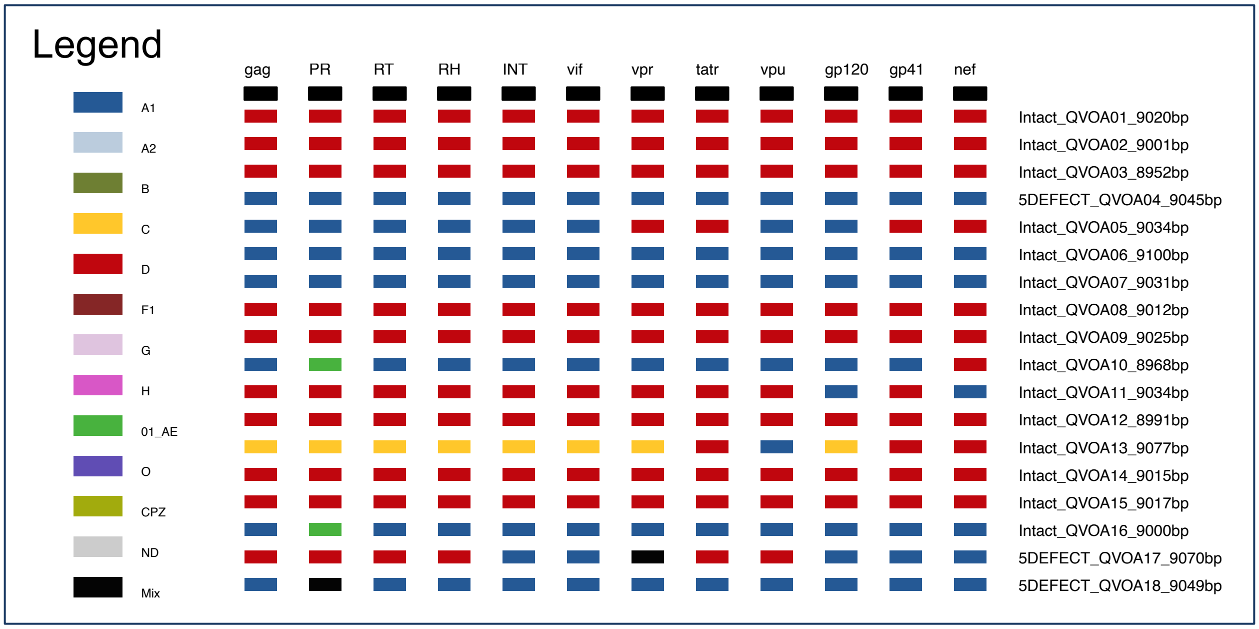

Figure S16. HIV-1 subtyping of the 18 QVOA-derived RNA genomes by MOCHI Proviral Subtyping Express 1.0 reveals that 5' defect was associated with both subtype A1 and D genomes at the 5' end.

This lack of pattern made it impossible to identify clear differences between intact and 5' defective sequences within this region, that could be leveraged to modify HIVSeqinR settings to better distinguish intact from 5' defective subtype A1 and D sequences. Future studies should sequence a much larger number of HIV genomes inclusive of the *psi* region from QVOA-derived replication competent viruses to better understand genotype/phenotype correlations in this region, towards this goal.

Taken together, we estimate that the original HIVSeqinR settings yield 83% sensitivity and the highest currently achievable level of specificity when classifying subtype A1 and D sequences, so we did not further modify any of its original parameters for the present study.

Given the incomplete knowledge of genotype/phenotype relationships in subtype A1 and D *psi* sequences however, HIVSeqinR may currently underestimate the level of genome-intactness if the *psi* region of a given genome has unique insertions and/or deletions. Given this incomplete knowledge, we elected to not manually over-ride individual calls in the present study. Nevertheless, we suggest that users applying HIVSeqinR to subtypes A1 and/or D consider their specific research objectives when

deciding whether to visually evaluate all genomes, and potentially manually over-ride selected HIVSeqinR calls.

References

1. Lee, G. Q. *et al.* HIV-1 DNA Sequence Diversity and Evolution during Acute Subtype C Infection. *Nat. Commun.* **10**, 2737 (2019).
2. Lee, G. Q. Chemistry and Bioinformatics Considerations in Using Next-Generation Sequencing Technologies to Inferring HIV Proviral DNA Genome-Intactness. *Viruses* **13**, (2021).
3. Lee, G. Q. & Lichterfeld, M. Near-Full-Length Single-Genome HIV-1 DNA Sequencing. *Methods Mol. Biol.* **2407**, 357–364 (2022).
4. Lee, G. Q. *et al.* Clonal expansion of genome-intact HIV-1 in functionally-polarized Th1 CD4 T cells. *J. Clin. Invest.* **127**, 2689–2696 (2017).
5. Einkauf, K. B. K. B. *et al.* Intact HIV-1 proviruses accumulate at distinct chromosomal positions during prolonged antiretroviral therapy. *J. Clin. Invest.* **129**, 988–998 (2019).
6. Kinloch, N. N. *et al.* HIV reservoirs are dominated by genetically younger and clonally enriched proviruses. *MBio* **14**, (2023).
7. Kuo, H.-H. *et al.* Blood and Lymph Node Dissemination of Clonal Genome-Intact HIV-1 DNA Sequences During Suppressive Antiretroviral Therapy. *J. Infect. Dis.* **222**, 655-660 (2020).
8. Jiang, C. *et al.* Distinct viral reservoirs in individuals with spontaneous control of HIV-1. *Nature* 1–7 (2020) doi:10.1038/s41586-020-2651-8.
9. Los Alamos National Laboratory. Los Alamos HIV sequence database. http://www.hiv.lanl.gov/.
10. Poon, A. F. Y. *et al.* Quantitation of the latent HIV-1 reservoir from the sequence diversity in viral outgrowth assays. *Retrovirology* **15**, (2018).
11. Lee, G. Q. *et al.* Prevalence and Clinical Impacts of HIV-1 Intersubtype Recombinants in Uganda Revealed by a Near-Full-Genome Population and Deep Sequencing Approaches. *AIDS* **31**, 2345–2354 (2017).
