# Supplementary material for: HIV-1 subtype A1, D, and recombinant proviral genome landscapes during long-term suppressive therapy
Source: Nat Commun. 2024 Jul 2;15:5480. doi: 10.1038/s41467-024-48985-9 (PMC11219899; doi:10.1038/s41467-024-48985-9)
Supplement: Supplementary file 3 — Reporting Summary [file 41467_2024_48985_MOESM3_ESM.pdf]

nature portfolio

Corresponding author(s): Guinivere Q. Lee

Last updated by author(s): Apr 23, 2024

Reporting Summary

Nature Portfolio wishes to improve the reproducibility of the work that we publish. This form provides structure for consistency and transparency in reporting. For further information on Nature Portfolio policies, see our Editorial Policies and the Editorial Policy Checklist.

Statistics

For all statistical analyses, confirm that the following items are present in the figure legend, table legend, main text, or Methods section.

n/a	Confirmed	
	x	The exact sample size (*n*) for each experimental group/condition, given as a discrete number and unit of measurement
	x	A statement on whether measurements were taken from distinct samples or whether the same sample was measured repeatedly
	x	The statistical test(s) used AND whether they are one- or two-sided<br>*Only common tests should be described solely by name; describe more complex techniques in the Methods section.*
x		A description of all covariates tested
x		A description of any assumptions or corrections, such as tests of normality and adjustment for multiple comparisons
	x	A full description of the statistical parameters including central tendency (e.g. means) or other basic estimates (e.g. regression coefficient) AND variation (e.g. standard deviation) or associated estimates of uncertainty (e.g. confidence intervals)
	x	For null hypothesis testing, the test statistic (e.g. *F*, *t*, *r*) with confidence intervals, effect sizes, degrees of freedom and *P* value noted<br>*Give P values as exact values whenever suitable.*
x		For Bayesian analysis, information on the choice of priors and Markov chain Monte Carlo settings
x		For hierarchical and complex designs, identification of the appropriate level for tests and full reporting of outcomes
	x	Estimates of effect sizes (e.g. Cohen's *d*, Pearson's *r*), indicating how they were calculated

Our web collection on statistics for biologists contains articles on many of the points above.

Software and code

Policy information about availability of computer code

Data collection	ddPCR data was collected using QuantaSoft (BioRad Laboratories, Inc., version 1.7.4).
Data analysis	Custom code HIVSeqinR version 2.7.1 was used to categorize HIV DNA sequences into "intact" or various types of "defective" viral genomes. We have noted in the manuscript text that the source code is freely available in GitHub (https://github.com/guineverelee/HIVSeqinR DOI: 10.5281/zenodo.10998708).<br>HIV-1 subtyping was performed using MOlecular Characterization of HIV-1 (MOCHI) Proviral Subtyping Express 1.0, an R-language based subtyping tool developed as part of this study specific for HIV-1 DNA subtype determination. We have noted in the manuscript text that the source code is freely available in GitHub (https://github.com/guineverelee/subtype_express/ DOI: 10.5281/zenodo.10998392).<br>ddPCR data was analyzed using QuantaSoft (BioRad Laboratories, Inc., version 1.7.4). Summary statistics and comparisons between groups were performed using GraphPad Prism 9 or R.

For manuscripts utilizing custom algorithms or software that are central to the research but not yet described in published literature, software must be made available to editors and reviewers. We strongly encourage code deposition in a community repository (e.g. GitHub). See the Nature Portfolio guidelines for submitting code & software for further information.

Data

Policy information about availability of data

All manuscripts must include a data availability statement. This statement should provide the following information, where applicable:

- Accession codes, unique identifiers, or web links for publicly available datasets
- A description of any restrictions on data availability
- For clinical datasets or third party data, please ensure that the statement adheres to our policy

All raw HIV sequence data that support the findings of this study have been deposited in GenBank (OQ686003 – OQ686609 and PP297232-PP297249). All other data supporting the findings of this study are available within the paper and its supplementary information files.

Research involving human participants, their data, or biological material

Policy information about studies with human participants or human data. See also policy information about sex, gender (identity/presentation), and sexual orientation and race, ethnicity and racism.

Reporting on sex and gender	This study was conducted in a region that does not allow for gender self-assignment.<br>Data on biological sex was collected by self-report. Of the 23 participants included, 16 (70%) were female.<br>This study examined HIV-1 proviral landscapes in samples collected from both males and females (16/23 were female, or 70%). However, methods used to assess intact and defective proviruses were not quantitative (FLIPseq is biased towards shorter reads), and therefore data is not presented disaggregated by participant characteristics.
Reporting on race, ethnicity, or other socially relevant groupings	This study was conducted in rural Uganda and all participants were ethnically Bugandan and racially Black. As all participants were of the same ethnicity and race, these socially relevant groupings are not distinguished in reporting or the analyses.
Population characteristics	Samples from 23 individuals with HIV-1 were included in this study. The HIV-1 subtype distribution of these individuals was A1 (n=6), C (n=1), D (n=11) and A1/D recombinant (n=5). The median age was 42 (min/max 29-53, IQR 38-45), and 16/23 (70%) were female. All participants were on suppressive antiretroviral therapy for HIV-1, and had been on therapy for a median of nine years (IQR 6-11). Participant median CD4 count at the time of sample collection was 761 cells/µL (IQR 586-892). Replication-competent HIV-1 reservoir sizes at sampling were a median of 0.5 (IQR 0.3-1.3) infectious units per million cells (IUPM), as measured by Quantitative Viral Outgrowth Assays (QVOA).
Recruitment	This was a nested retrospective study using previously banked samples. No new recruitment was performed for this study.
Ethics oversight	The study was approved by the National Institute of Allergy and Infectious Diseases (National Institutes of Health), Uganda Virus Research Institute, Uganda National Council for Science and Technology, and Weill Cornell Medicine Institutional Ethics Review Board (20-01021318). Reservoir quantification in subtype B samples was approved by the University of British Columbia/Providence Health Care and Simon Fraser University Research ethics boards. All participants provided written informed consent.

Note that full information on the approval of the study protocol must also be provided in the manuscript.

Field-specific reporting

Please select the one below that is the best fit for your research. If you are not sure, read the appropriate sections before making your selection.

☒ Life sciences ☐ Behavioural & social sciences ☐ Ecological, evolutionary & environmental sciences

For a reference copy of the document with all sections, see nature.com/documents/nr-reporting-summary-flat.pdf

Life sciences study design

All studies must disclose on these points even when the disclosure is negative.

Sample size	No sample size calculation was performed. Of n=90 virally-suppressed individuals with HIV, n=23 were selected for near-full-length HIV-1 DNA genome sequencing to give a representation of the cohort's sex and HIV subtype distribution. This approach was chosen due to the cost and labor requirements of this assay.
Data exclusions	No data were excluded from the analyses.
Replication	Every viral PCR and sequencing run was performed with a positive control (lab strain HIV with known sequence data) to monitor sequencing quality, as well as a negative control to guard against PCR contamination. Positive and negative controls were also included in every ddPCR experiment (IPDA-B, IPDA-A1D). A minimum of three technical replicates were performed for each sample, and all ddPCR plots were visually inspected during data analysis to ensure that results of all replicates were consistent with each other.
Randomization	This was an observational study and there was no allocation to experimental groups.

Blinding	This was an observational study and there was no allocation to experimental groups.

Reporting for specific materials, systems and methods

We require information from authors about some types of materials, experimental systems and methods used in many studies. Here, indicate whether each material, system or method listed is relevant to your study. If you are not sure if a list item applies to your research, read the appropriate section before selecting a response.

Materials & experimental systems

n/a	Involved in the study
x	Antibodies
	x Eukaryotic cell lines
x	Palaeontology and archaeology
x	Animals and other organisms
x	Clinical data
x	Dual use research of concern
x	Plants

Methods

n/a	Involved in the study
x	ChIP-seq
x	Flow cytometry
x	MRI-based neuroimaging

Eukaryotic cell lines

Policy information about cell lines and Sex and Gender in Research

Cell line source(s)	J-Lat 9.2 cell line: obtained from the NIH AIDS Reagent Program (catalogue number: 9848), Division of AIDS, NIAID, NIH<br>J-Lat 10.6 cell line: obtained from the NIH AIDS Reagent Program (catalogue number: 9849), Division of AIDS, NIAID, NIH
Authentication	J- Lat 9.2 and 10.6 cell lines: The expected HIV:Cell 1:1 ratio was observed in ddPCR experiments performed in this manuscript.
Mycoplasma contamination	Tests for bacteria, fungi and mycoplasma were negative according to the NIH AIDS Reagent Program for both J-Lat cell lines.
Commonly misidentified lines<br>(See ICLAC register)	none used

Plants

Seed stocks	n/a
Novel plant genotypes	n/a
Authentication	n/a
